# Application of Homology Modeling by Enhanced Profile–Profile Alignment and Flexible-Fitting Simulation to Cryo-EM Based Structure Determination

**DOI:** 10.3390/ijms23041977

**Published:** 2022-02-10

**Authors:** Yu Yamamori, Kentaro Tomii

**Affiliations:** 1 Artificial Intelligence Research Center (AIRC), National Institute of Advanced Industrial Science and Technology (AIST), 2-4-7 Aomi, Koto-ku, Tokyo 135-0064, Japan; yu.yamamori@aist.go.jp; 2 AIST-Tokyo Tech Real World Big-Data Computation Open Innovation Laboratory (RWBC-OIL), National Institute of Advanced Industrial Science and Technology (AIST), 2-4-7 Aomi, Koto-ku, Tokyo 135-0064, Japan

**Keywords:** cryo-EM, flexible fitting, homology modeling, profile–profile alignment

## Abstract

Application of cryo-electron microscopy (cryo-EM) is crucially important for ascertaining the atomic structure of large biomolecules such as ribosomes and protein complexes in membranes. Advances in cryo-EM technology and software have made it possible to obtain data with near-atomic resolution, but the method is still often capable of producing only a density map with up to medium resolution, either partially or entirely. Therefore, bridging the gap separating the density map and the atomic model is necessary. Herein, we propose a methodology for constructing atomic structure models based on cryo-EM maps with low-to-medium resolution. The method is a combination of sensitive and accurate homology modeling using our profile–profile alignment method with a flexible-fitting method using molecular dynamics simulation. As described herein, this study used benchmark applications to evaluate the model constructions of human two-pore channel 2 (one target protein in CASP13 with its structure determined using cryo-EM data) and the overall structure of *Enterococcus hirae* V-ATPase complex.

## 1. Introduction

The application of cryo-electron microscopy (cryo-EM) has become an important tool for ascertaining the structures of large biomolecules such as ribosomes and membrane protein complexes [1,2,3,4,5,6,7,8]. Its achievements are underscored by the recent increase in the number of Protein Data Bank (PDB) archive entries related to cryo-EM [9]. Although improvements in both cryo-EM technology and software enable us to obtain near-atomic-resolution EM data [4], it is likely that only a medium-to-low resolution density map is obtained, either partially or completely. Methods must be examined to bridge the gap separating weak density derived from cryo-EM experiments and an atomic-resolution model.

For the determination of atomic structures, various methods have been proposed based on density maps derived from cryo-EM experiments. Two important categories are template-free, i.e., de novo modeling methods [10,11,12,13,14] and template-based, i.e., flexible-fitting modeling methods [15,16,17,18,19,20,21]. De novo modeling is used for methods constructing an all-atom or Cα model by detecting and tracing atomic positions in an EM map without using a template structure. For example, de novo modeling with the pathwalking method uses a traveling salesman problem solver for backbone tracing, which requires slight manual adjustment [10,11]. The Rosetta software suite includes an automatic de novo modeling protocol, which assembles fragment structures from a protein-structure library with subsequent optimization, to be fitted to an EM map [12,13]. Another recently developed fully automatic method, MAINMAST, is a method connecting local dense points identified as main-chain or side-chain positions by presuming a minimum spanning tree. EM maps with around 4 Å resolution have sufficient information for this method [14]. Flexible-fitting modeling methods optimize an initial structure to achieve a better fit to an EM map using various methods such as real-space refining [15,22], molecular dynamics (MD) simulation [16,18,19,20,21], and normal mode analysis [17]. Initial structures are provided from known X-ray structures [17] or are based on the results of homology modeling [18]. MDFF, which introduces an EM-map-derived term into the MD force field, has been a widely applied method among flexible-fitting methods [20,23,24]. Other methods such as cryo-fit [21], cryo-fit2 [25], and MDfit [19] are classified as cross-correlation-based methods in which a bias term to the EM map is added to the force field. This is designed to maximize the cross-correlation coefficient (CCC) [17,26] between an EM map and an atomic model.

Regarding recent progress in the modeling of protein structures, AlphaFold2 (AF2) [27] achieved magnificent results in the modeling of a monomer structure of protein for comprehensive targets in CASP14 [28]. For multimer prediction, it remains a frontier of prediction. For CASP14, a method that combines template-based and ab initio docking with deep-learning-based contact prediction has shown great success for multimer prediction [29,30,31]. In fact, AF2, in its earlier versions, did not support multimer prediction. It is not necessarily a good initial structure for rigid-body docking, even though the prediction was regarded as a success for a monomer structure [32]. In more recent progress, AF2-multimer [33] was tuned for multimer prediction. Other efforts to apply the original AF2 to multimer prediction have been reported in the relevant literature [34,35]. Of course, these also led to considerable improvement in multimer prediction. Research does not seem to have reached the stage at which AF2 has achieved its monomer predictions. For application to cryo-EM-data-based modeling, recent CASP14 indicates that examples exist for which the AF2 model improves the accuracy of the structure determined using the de novo method [36]. More recently, AF2 has been applied to a more practical case of modeling of nuclear pore complexes [37].

As an application of our developed homology modeling method [38], we propose a new scheme of atomic structure modeling based on a cryo-EM density map with subnanometer or higher resolution. We have developed a highly sensitive and accurate homology modeling method using enhanced profile–profile alignments. The profile–profile alignment method has been widely regarded as a powerful tool not only for finding proteins suitable for templates but also for producing an accurate alignment between a target sequence and a template sequence. Our own profile–profile alignment and comparison methods, constituting the FORTE method uses a scoring scheme based on the correlation coefficient between two profile columns [39,40]. Modeling protocols including profile–profile alignment using FORTE have already been applied to many advanced or practical studies: past and recent critical assessments of techniques for protein structure predictions (CASP) experiments [38,40], critical assessment of predicted interactions (CAPRI) experiments [41,42], supporting the structure determination and modeling of the sorting and assembly machinery (SAM) complex [5], translocation of the outer membrane (TOM) complex (mitochondrial outer membrane translocators) [43], and a CASP community-wide experiment on modeling SARS-CoV-2 proteins causing the coronavirus disease, CASP-commons (CASP-COVID) [44]. The noteworthy properties of our method are its ability to predict oligomeric structures and its suitability for providing an initial structure for structure determination using cryo-EM [9,38]. For the study described herein, we combine our homology modeling method using enhanced profile–profile alignments and flexible fitting using MD simulation. The homology models generated using our method and filtered by the degree of agreement with an EM map are used as initial structures for flexible-fitting simulation. We expect that there are conditions under which our homology models can be better starting points for flexible-fitting simulation than models from other methods. These include the condition that the target is a multimer and that the provided EM map has low resolution that is inapplicable to de novo modeling.

To demonstrate the performance and applicability of our method, we present results obtained for two applications intended for modeling of a protein complex for which structures are already known and for one application of modeling the protein complex for which the structure has not yet been determined: human two-pore channel (TPC) 2 and the whole structure of *Enterococcus hirae* V-ATPase (*Eh* V-ATPase). Human TPC2 is a target in CASP13 for which structures have been ascertained using the cryo-EM technique [9,45]. Furthermore, in the Supporting Information, we present an additional example of modeling for the a-subunit of *Thermus thermophilus* V-ATPase, with a structure that was determined using cryo-EM techniques. For comparison of the performance of our homology modeling method with those of other methods as the generator of a starting point for flexible fitting, flexible-fitting simulations were undertaken for all predicted models of the target, generated without information from the cryo-EM map. Then, the entire structure of *Eh* V-ATPase, for which the atomic structure remains unclear, is presented as a demonstration that our method is applicable for a huge and complex target. Until recently, cryo-EM map single-particle analysis with the Zernike phase plate provided the entire structure of *Eh* V-ATPase only at 17.3 Å resolution [46]. In this report, we explain the construction of atomic structure models of *Eh* V-ATPase based on the density map from a further improved cryo-EM map at subnanometer resolution using the Volta phase plate. Our method generated a reasonable model of the entire structure of *Eh* V-ATPase that agrees well with the EM map.

## 2. Materials and Methods

Our proposed method includes two parts: generating a set of models using our homology modeling method for a target sequence or sequences, and flexible fitting of the generated models via an MD simulation biased to an EM map. First, we generate a set of three-dimensional (3D) models through our template-based modeling pipeline. These models are evaluated both by the score based on fitting with the EM map and the scores of model quality. Then, models with higher scores are subjected to flexible-fitting simulations to improve the fit to the EM map. Finally, scores that evaluate the quality of the 3D models and the fitting with the EM map are used as criteria for selecting models. For protein-complex modeling, the EM map is divided into fragments corresponding to each subunit. Then, 3D models generated by the homology modeling pipeline for each chain (subunit) are evaluated based on fitting with the EM map fragments. Models with higher scores for each subunit are used for flexible fitting of the whole model.

### 2.1. Profile–Profile-Alignment-Based Homology Modeling

**Figure 1:**
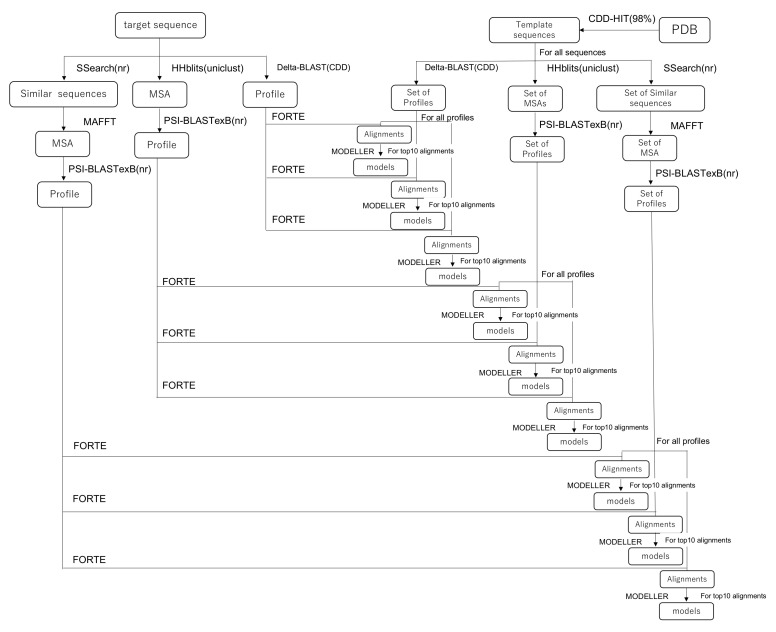
Schematic showing our homology modeling pipeline.

The homology modeling method is based on the latest version of our pipeline [38]. The whole protocol of the homology modeling method is summarized as the pipeline in Figure 1, which presents the following steps: (1) profile constructions, (2) profile–profile alignment and scoring, (3) 3D model construction, and (4) evaluations of models in both senses of scoring functions and the fit to the EM map.

First, the step for the construction of profiles is explained. Technical details are presented in the Supporting Information of the references [38]. As the template library of sequences, a representative set of protein chain sequences was extracted from PDB using CD-HIT [47,48] with the threshold of 98 % sequence identity. Three sequence retrieval methods were applied for both target and template sequences. (1) In the first method, we used SSEARCH [49] to obtain similar protein sequences with a sensitive matrix MIQS [50] against the NCBI nr database. Then, a multiple sequence alignment (MSA) was obtained using MPI-parallelized MAFFT [51]. To construct a profile, PSI-BLASTexB [52], an extension of the original PSI-BLAST [53], was used to obtain a better position-specific scoring matrix. (2) In the second method, we conducted a DELTA-BLAST [54] with one iteration against the NCBI’s Conserved Domain Database (CDD) to construct a profile. (3) In the third method, an HHblits [55] search was first performed against the Uniclust database [56] to find similar protein sequences and an MSA using them. Then, we performed a PSI-BLASTexB search against the NCBI’s nr database using the constructed MSA as a seed MSA. The combination of software packages and databases used for the profile generation are summarized in Table 1.

FORTE, our profile–profile alignment algorithm, was used to calculate an alignment and a score between a target and template profiles. The FORTE scoring scheme is based on the correlation coefficient found between the two profile columns to be compared. The Z-scores of the alignments are calculated using alignment scores and log-length correction. If no good template is found for the entire length of the target sequence in this step, then the target sequence is split into multiple fragments, where possible, in order to find a good template.

Based on the obtained alignments of the target sequence with the template sequence and their Z-scores, the 3D models of the target protein (or protein complex) were constructed using MODELLER [57]. We constructed 3D models with higher-ranked templates according to their Z-scores. Fundamentally, for each combination of query profile and template profiles, the 10 highest-ranked alignments generated by FORTE were used for model construction. For each alignment, five models were constructed by MODELLER. If several good templates existed, then we also tried to generate models with multi-template alignment. In the construction of the multimer, when multimer templates were found, those templates were assigned priority. The generated models were sorted by the model quality scores calculated using the VERIFY3D [58,59] and dDFIRE programs [60,61]. The secondary structure of a target protein was also predicted using RaptorX Property [62], which is an effective method. The available standalone scripts can be implemented easily in our modeling pipeline. Similarity between the secondary structure of a model and the predicted structure was evaluated at this stage. All constructed models except for those with an extremely low score were subjected to a series of rigid-body dockings to the EM map. The CCC of each model was calculated. Rigid-body docking and calculation of the CCC was performed using the colores program in the Situs package [63]. About ten up to hundreds of models with higher scores were selected as candidates for the next step: flexible fitting. Among the described criteria, we preferred the goodness-of-fit of the CCC between the model and the EM maps to the CCCs found for others.

### 2.2. Correlation-Based Flexible Fitting

Flexible fitting is aimed at fitting an atomic model to the density map derived from a cryo-EM experiment. In our scheme, we adopted the cryo-fit [21] or cryo-fit2 program [25] in PHENIX. For TPC2, flexible-fitting simulations were performed using cryo-fit2 with the respective conditions of map-weight-multiply =100. For *Eh* V-ATPase, flexible-fitting simulations were performed using cryo-fit with the MD engine, a modified version of GROMACS [64].

Hereinafter, we briefly summarize the method we adopted. The flexible-fitting protocol is fundamentally CCC-based fitting using MD simulation. This fitting was performed to try to maximize CCC without loss of structural features of the models such as secondary structure or residue–residue contacts. A benefit of CCC is that it is a measure of how the model fits a cryo-EM density map [17,26]. The density derived from cryo-EM is represented by an intensity vector on a cubic lattice as $\rho^{\exp.}\left(i,j,k\right)$, where (i,j,k) are the indices for grid space points. To measure the similarity between an atomic model and a cryo-EM density map, the computed density corresponding to the atomic coordinates of the model is defined as

(1) $$\rho^{\mathrm{sim}.}=\sum_{n=1}^{N}\int \int \int_{V_{ijk}}g_{n}\left(x,y,z\right)dxdydz.$$

In addition, the Gaussian function of (x,y,z) space $g_{n}\left(x,y,z\right)$ is defined as

(2) $$g_{n}\left(x,y,z\right)=\exp\left[-\frac{3}{2\sigma^{2}}\left\{{\left(x-x_{n}\right)}^{2}+{\left(y-y_{n}\right)}^{2}+{\left(z-z_{n}\right)}^{2}\right\}\right],$$

where $\left(x_{n},y_{n},z_{n}\right)$ denote the coordinates of the *n*-th atom of the model and σ represents the cryo-EM map resolution. Then, the CCC between the cryo-EM data and atomic models is defined as

(3) $$CCC=\frac{\sum_{ijk}\rho^{\exp.}\left(i,j,k\right)\rho^{\mathrm{sim}.}\left(i,j,k\right)}{\sqrt{\sum_{ijk}\rho^{\exp.}{\left(i,j,k\right)}^{2}\rho^{\mathrm{sim}.}{\left(i,j,k\right)}^{2}}}.$$

The objective function of the flexible-fitting simulation includes the biased term $E_{\mathrm{EM}}$ to maximize CCC as

(4) $$E_{\mathrm{EM}}=N\times k\left(1-CCC\right),$$

where *k* is a parameter for the strength of the biased term along with the force field term and *N* stands for the number of heavy atoms. Furthermore, an additional term to maintain the residue–residue contact of the initial structure is used.

### 2.3. Correlation-Based Metrics

For evaluation of the model in the terms of the goodness-of-fit to the EM map, cross-correlation-related (CC-related) values implemented in the map_model_cc module in PHENIX such as CC${}_{\mathrm{mask}}$, CC${}_{\mathrm{peaks}}$, and CC${}_{\mathrm{volume}}$ [65] were used as a measure of overall fitness. CC${}_{\mathrm{mask}}$ uses map values inside a mask with a fixed radius to measure the fit of atomic centers. In addition, CC${}_{\mathrm{volume}}$ and CC${}_{\mathrm{peaks}}$ compare the map regions with the highest density values to to measure the respective degrees of fit of molecular envelopes and the strongest peaks [22]. PHENIX’s local CC${}_{\mathrm{box}}$ measure and the segment-based Manders’ overlap coefficient (SMOC) score [66] implemented in the TEMPy software [67] were used as measures of the local model-to-map fitness. Selection of these measures was conducted based on correlation analyses of various measures related to the fit to an EM map [9].

### 2.4. Modeling of human TPC2 (T0984o in CASP13)

For demonstration and comparison of the performance of our method with others, T0984o from CASP13 was selected [9]. Human TPC2 T0984o is a homodimer complex with 752 residues per monomer. Two-pore calcium channel proteins play important roles in regulating lysosomal membrane potential. The apo structure was found using cryo-EM techniques based on an EM map with 3.5 Å resolution (PDB ID code is 6NQ1; EMDB ID code is EMD-0478) [45]. In CASP13, for the target T0984o, all submitted models were generated without information from cryo-EM data (the target deadline in CASP13 was 27 June 2018); 157 dimeric structures submitted from 34 groups including ours are available from the CASP13 website (https://www.predictioncenter.org/casp13/multimer_results.cgi?target=T0984o, accessed on 10 January 2022). Our five submitted models were generated using the homology modeling pipeline explained herein. The PDB ID codes of the template used were 6C96 and 6C9A (both are homodimer structures) [68]. First, all the submitted models were subjected to rigid-body fitting to the EM map. Then, after excluding the models that fit the EM map poorly, flexible-fitting simulations of the 96 models were performed. After flexible fitting, real-space-refinement calculations using PHENIX were performed. Finally, a comparison between our fitted model and the PDB structure was performed in terms of both the goodness of fit to the EM map and the similarity with the reference structure. The metrics described in Section 2.3 were used for the goodness of fit to the EM map. The TM-score [69] was used for measuring similarity among structures.

### 2.5. Modeling of *Eh* V-ATPase

**Figure 2:**
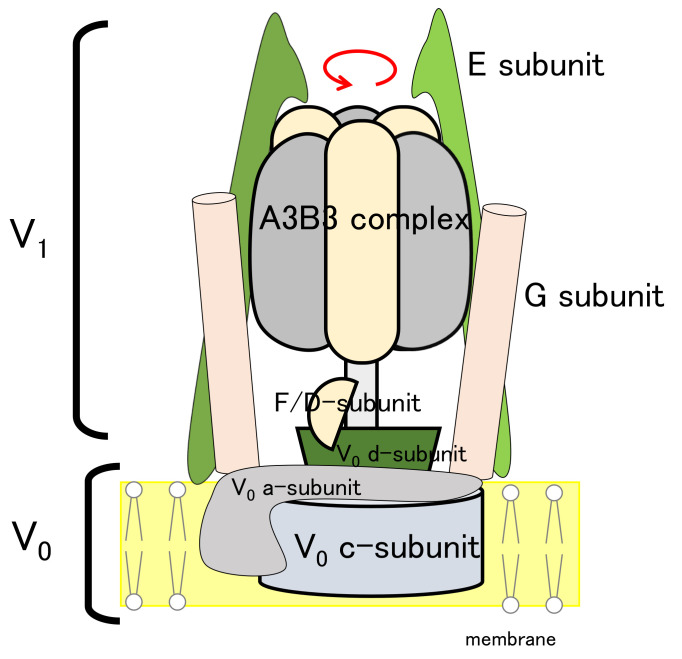
Schematic view of *Eh* V-ATPase.

As a demonstration of our method for a rather practical case, the target biomolecule of *Eh* V-ATPase was used. V-ATPase is a rotary molecular motor that actively transports ions coupled with ATP hydrolysis. It comprises 24 chains with 9 types of subunits (three A-subunits, three B-subunits, a D-subunit, two E-subunits, an F-subunit, two G-subunits, an a-subunit, 10 c-subunits, and a d-subunit), as shown in Figure 2. The membrane-embedded region, called the V${}_{0}$ region (a-, c-, and d-subunits), functions for ion transportation. The water-soluble region, designated as the V${}_{1}$ region (A-, B-, D-, E-, F-, and G-subunits), functions for ATP hydrolysis. The two EG complexes are called stalk A and B. In comparison with the well-studied molecular motor F-ATPase, there are counterpart subunits except for the d-subunit in the V${}_{0}$ region of V-ATPase. Both V-ATPase and F-ATPase have been known to rotate 120 degrees counterclockwise per ATP hydrolysis. Very recent studies of single-molecule analysis of *Eh* V${}_{0}$ ATPase revealed a sub-step at 40 degrees. It is noteworthy that the sub-step differs from those of the well-studied F1-ATPase. Some subunits have already been found using X-ray crystallography, such as the 10-mer of the c-subunit complex c-ring (PDB ID code: 2BL2 [70]), DF-complex (PDB ID code: 3AON [71]), A3B3-complex (PDB ID code: 3VR2 [72]), and A3B3DF-complex (PDB ID code: 5KNB [73]). The first entire structure appeared recently at 17.3 Å resolution using cryo-EM data for recombinant *Eh* V-ATPase (EMDB ID code: EMD-9661 [46]). Here, we particularly examine the construction of an atomic model based on cryo-EM data at 6.5 Å resolution, derived from cryo-EM experiments with the Volta phase plate provided by the National Institute for Physiological Sciences (NIPS) Electron Microscopy Group.

The following strategy for modeling of *Eh* V-ATPase was used. First, the EM map was split into subunits with appropriate size using UCSF Chimera [74,75]. For subunits where individual structures had already been found using X-ray observations, rigid-body fitting to the EM map was performed (c-ring and A3B3DF complex). Further flexible-fitting MD simulation was performed for the A3B3DF-complex. In cases for which the whole subunit structure is unknown, homology modeling was performed (E-, G-, a-, and d-subunits). The most recent date of the considered template was published as February 2019. For this stage, we prepared multiple models and ranked them based on how they fit the EM map for each subunit. Then for higher-ranked models, flexible fitting was performed using MD simulation with restraints for the EM map. Finally, each subunit model was assembled. For placement of the E- and G-subunits as stalk A or B, we first used a model following the arrangement of 5Y5X [76]. Other subunits were assembled based on cryo-EM maps and partially reconstructed using MODELLER to avoid clashes. Further overall flexible fitting was performed using MD simulation. The MolProbity [77] score and the CCC with the whole EM map were calculated for the models at the final stage.

## 3. Results

### 3.1. Modeling of human TPC2 (T0984o in CASP13)

**Figure 3:**
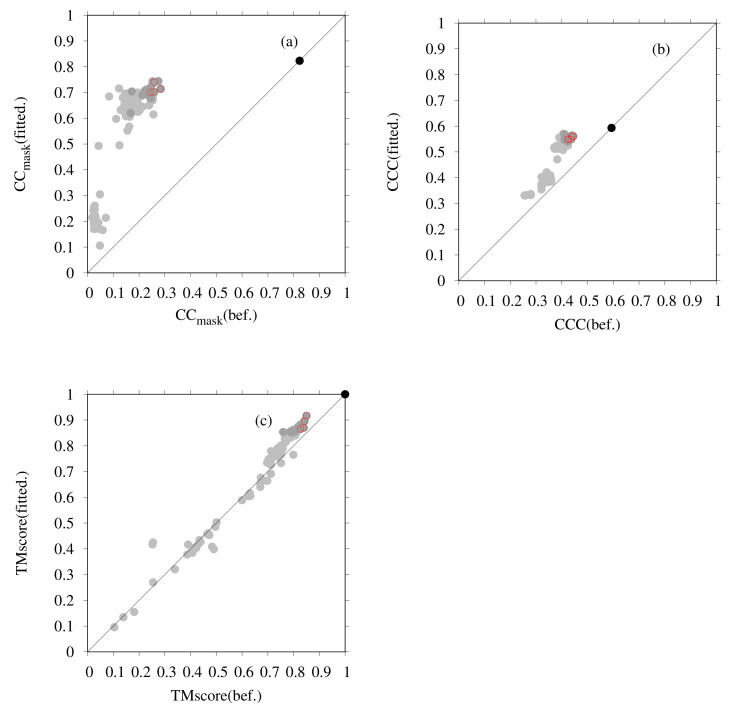
Flexible-fitting simulation of submitted dimeric models to T0984o in CASP13: (**a**) changes in the values of CC${}_{\mathrm{mask}}$ (PHENIX) with EM map (EMD-0478) caused by the flexible-fitting simulation; (**b**) changes in the values of CCC (colores) caused by the flexible-fitting simulation; and (**c**) changes in the values of TM-score with the PDB structure (6NQ1) caused by the flexible-fitting simulation. For each figure, the X-axis shows the values of submitted models before fitting; the y-axis shows the values of models after fitting. Red points correspond to our five submitted models of D-Haven(TS329) (T0984TS329_o1, T0984TS329_o2, T0984TS329_o3, T0984TS329_o4 and T0984TS329_o5). Dark-gray points correspond to YASARA(TS004), Bates-DMM(TS163), Chou-u(TS047), Kiharalab-capri(TS303), Zhiping-Weng(TS114), Cabonelab(TS299), and Seok(TS068). Gray points correspond to other submitted models. The black point corresponds to the PDB structure.

Figure 3 presents the results of flexible fitting of all submitted dimer models for T0984o to the EM map (EMD-0478). In Figure 3, each graph shows the change in values of the fit to the EM map and shows the similarity to the reference structure for the submitted models and the models after fitting. In Figure 3a, the change in CC${}_{\mathrm{mask}}$ was calculated by PHENIX, in Figure 3b the change in CCC was calculated using colores, and in Figure 3c the changes in TM-scores are shown. The X-axis and Y-axis show values for the model obtained before and after fitting, respectively. TM-scores between the PDB structure (6NQ1) and model structures were calculated using the MM-align program [78]. One point, in the figures, represents one model. The black, red, and gray points correspond to the PDB structure, our five submitted models, and models submitted by other groups, respectively. Among the submitted models, our models, (before fitting) are top-ranked in terms of CC-related values and TM-score, as shown [9]. However, even for a model with a high TM-score, the CC${}_{\mathrm{mask}}$ value is rather lower (up to 0.3) than that of the PDB structure (0.8). From Figure 3, it is apparent that the CC-related values increased considerably and that the TM-score increased slightly as a result of the flexible-fitting simulation. Models with TM-score values >0.85 after the fitting simulation, such as YASARA(TS004), Bates-DMM(TS163), Chou-u(TS047), Kiharalab-capri(TS303), Zhiping-Weng(TS114), Cabonelab(TS299), and Seok(TS068), shown as dark-gray points in Figure 3, are also top-ranked.

**Figure 4:**
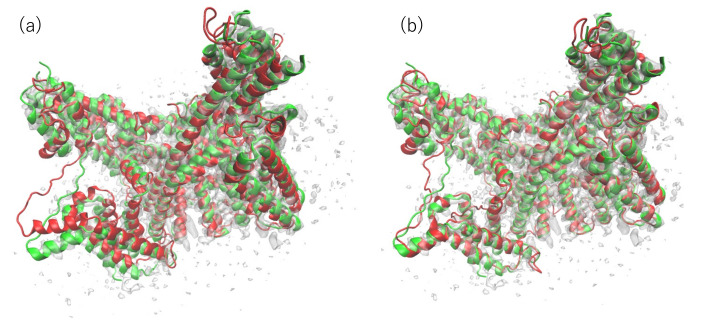
(**a**) 6NQ1 (green) and one of our submitted models (red) to T0984o in EM map (EMD-0478). The model is the rigid-body fit to the map produced by colores in the Situs program. (**b**) 6NQ1 (green) and our submitted model with the highest CC${}_{\mathrm{mask}}$ value after flexible-fitting simulation (red) to T0984o in the EM map (EMD-0478). The RMSD value of the fitted model with the reference PDB structure is 2.93 Å for the 1242 residues (except the missing residues of 6NQ1) (calculated using the MM-align program).

The superimposed structures between the PDB structure and our model with the highest CCC and TM-score (before and after fitting) are portrayed in Figure 4. The TM-score of this model is the highest among all the models subjected to flexible fitting. For consideration of the overall fit to the EM map, the CC-related values are presented for comparison in Table 2. These results demonstrate that our final model resembles the reference structure in terms of both the goodness of fit to the EM map and in terms of similarity in structure.

**Figure 5:**
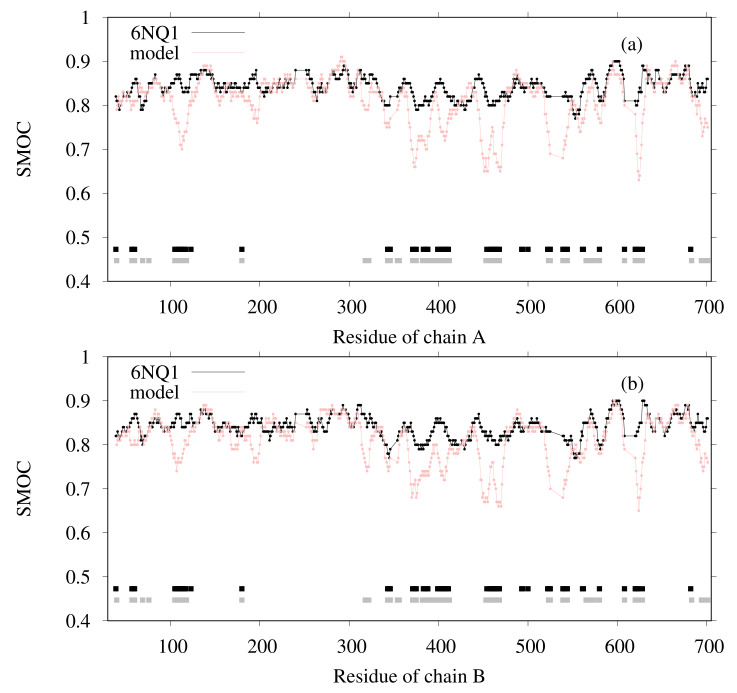
SMOC calculated using TEMPy for 6NQ1 and our model for (**a**) chain A and (**b**) chain B. The black line corresponds to 6NQ1. The pink line corresponds to our model. The black and gray lines show the residue region for which the template structures do not match well to the correspondence of the reference structure ( black line for 6C96; gray line for 6C9A).

**Figure 6:**
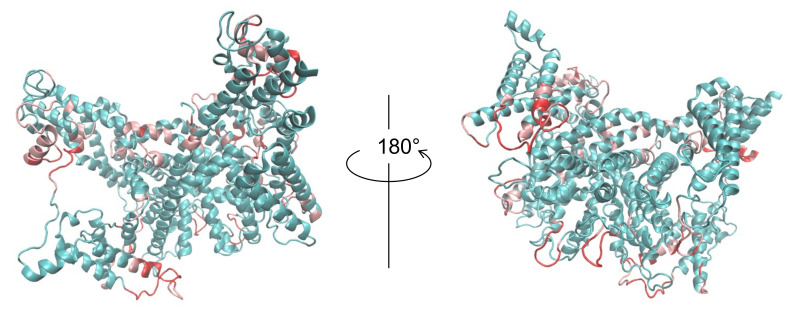
Our model with the highest CCC after flexible-fitting simulation. Regions in which the SMOC value of our model was lower than the value of 6NQ1 are highlighted as pink and red ( red regions are lower than pink).

The SMOC of each chain was also calculated for both the reference structure and for our model (Figure 5), indicating that our model agrees well with the EM map locally, except for several regions. The regions in which the SMOC value of our model is lower than the value of the reference structure are highlighted in pink and red (red regions are lower than pink) in Figure 6, except for the residues with almost equal values. For chain A, the residues 103–128, 190–198, 316–321, 366–410, 444–473, 521–525, 539–545, 558–565, 621–631, and 693–701 are the corresponding residues. For chain B, the residues 103–114, 190–199, 313–323, 367–392, 399–409, 444–472, 521–525, 539–546, 621–631, and 693–701 are the corresponding residues. To clarify this issue, we investigated the effects of template structures (PDB IDs are 6C96 and 6C9A) for our model. The similarity between template PDB structures and the reference PDB (6NQ1) structure was calculated using the TM-score program. In Figure 5, the regions wherein the template structures do not match the reference structure well are shown as bold lines on the bottom of the graphs (black line for 6C96 and gray line for 6C9A). For residue regions 106–116, both templates used lacked the corresponding region. These results suggest that, for some loop regions and regions near loops, the initial structure was insufficient to match the EM map, even after flexible-fitting simulation. This finding means, in other words, that the difficulty in the initial structure originated from the template structure.

### 3.2. Modeling of *Eh* V-ATPase

The results for the modeling of each subunit and the whole structure of *Eh* V-ATPase using our homology modeling pipeline and flexible fitting are examined. The structures of the complex of A- and B-subunits (A3B3-complex), complex of A-, B-, D-, and F-subunits (A3B3DF-complex or V${}_{1}$-center complex), and complex of c-subunits (10mer of c-subunit, c-ring) were found using X-ray crystallography. The structures of other subunits (E-, G-, a-, and d-subunits) remain unknown, therefore remaining as targets of the proposed method.

**Figure 7:**
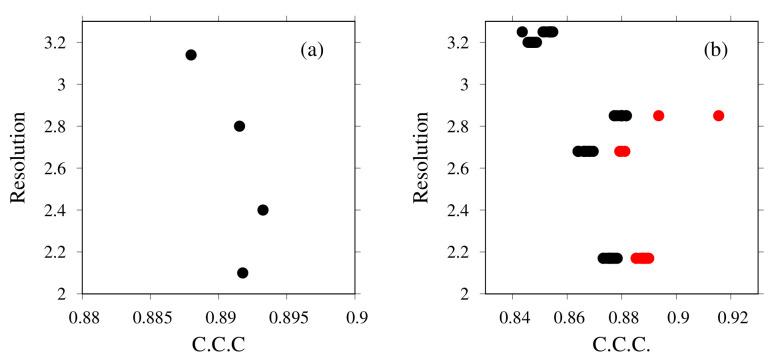
Rigid-body fitting of (**a**) c-ring and (**b**) V${}_{1}$-center complex (black points) and (**b**) flexible fitting of V${}_{1}$-center complex (red points). The X-axis shows the cross-correlation coefficient of crystal structures. The Y-axis shows the resolution for each structure.

We first examine how existing structures fit to the EM map (fragments). Figure 7a presents the results of rigid-body fitting to the corresponding EM map fragment of the X-ray structures of the c-ring. The X-axis presents the CCC values calculated using the colores program in the Situs package; the Y-axis shows the value of the resolution of the structure. Each black point corresponds to one structure, with PDB ID codes of 2BL2(2.1 Å), 2CYD(2.8 Å), 2DB4 [79] (2.4 Å), and 3AOU [79] (3.14 Å) (with numerical values in parentheses representing their resolutions). Considering both CCC and the resolution, the structure of 2BL2, with a CCC of 0.89, was selected for the next step.

Figure 7b presents the results of rigid-body fitting and flexible fitting of the V${}_{1}$-center complex. The sources of the initial structures of the V${}_{1}$-center complex are X-ray structures: PDB ID codes 3VR4 [72] (2.2 Å), 3VR5 [72] (3.9 Å), 3VR6 [72] (2.7 Å), 5KNB(3.3 Å), 5KNC [73] (3.0 Å), and 5KND [73] (2.9 Å). In addition to these, the docking structures of 3VR2(2.8 Å) and 3AON(2.0 Å), 3VR3(2.8 Å), and 3AON(2.0 Å) were considered. For each structure, residues, mainly short loops, without template(s) were modeled using MODELLER. The black points are rigid-body fitted structures. Models with high CCCs were subjected to flexible-fitting simulation. The red points are flexibly fitted structures of selected structures. For the next step, the flexibly fitted structure from 5KNB was selected. Its CCC was 0.92.

**Figure 8:**
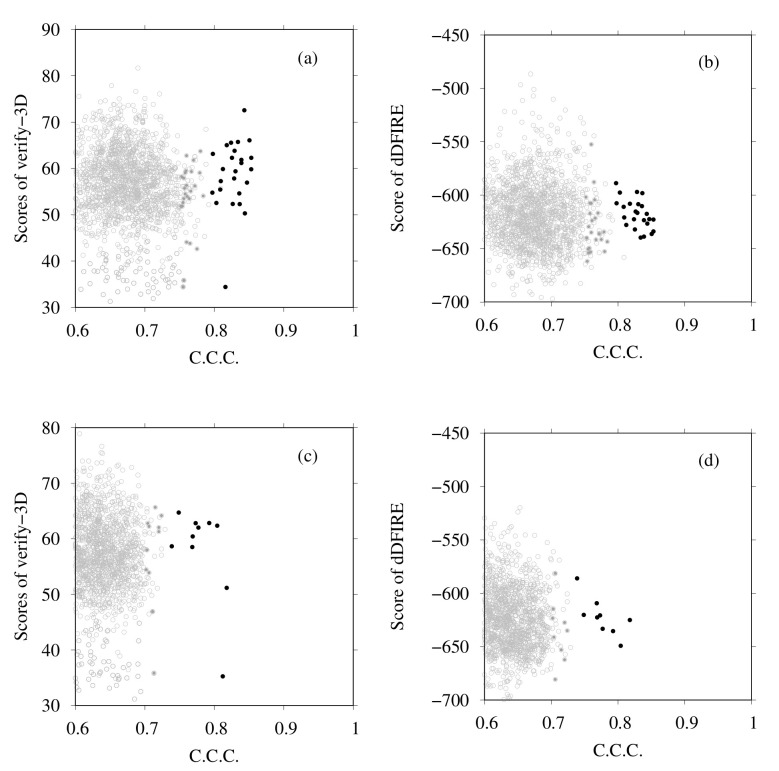
(**a**) Scores of VERIFY3D vs. CCC calculated using the colores of homology models before and after flexible-fitting simulations of EG complex (stalk A). (**b**) Scores of dDFIRE vs. CCC calculated using the colores of homology models before and after flexible-fitting simulations of EG complex (stalk A). (**c**) Scores of VERIFY3D vs. CCC calculated using the colores of homology models before and after flexible-fitting simulations of EG complex (stalk B). (**d**) Scores of dDFIRE vs. CCC calculated using the colores of homology models before and after flexible-fitting simulations of EG complex (stalk B). For each figure, the gray points denote homology models before flexible fitting. The filled dark-gray points represent the models selected for flexible fitting. The black points stand for models after flexible fitting.

**Figure 9:**
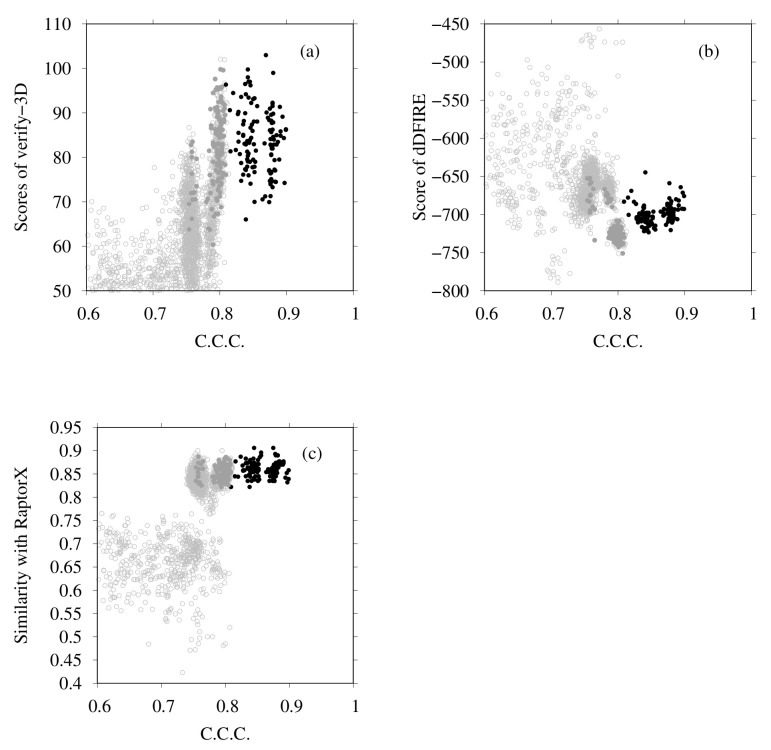
(**a**) Scores of VERIFY3D vs. CCC calculated using the colores of homology models before and after flexible-fitting simulations of the d-subunit. (**b**) Scores of dDFIRE vs. CCC calculated using the colores of homology models before and after flexible-fitting simulations of the d-subunit. (**c**) Similarity with secondary structure prediction by RaptorX Property vs. CCC calculated using the colores of homology models before and after flexible-fitting simulations of the d-subunit. For each figure, the gray points represent homology models before flexible fitting. The filled dark-gray points represent the models selected for flexible fitting. The black points denote models after flexible fitting.

**Figure 10:**
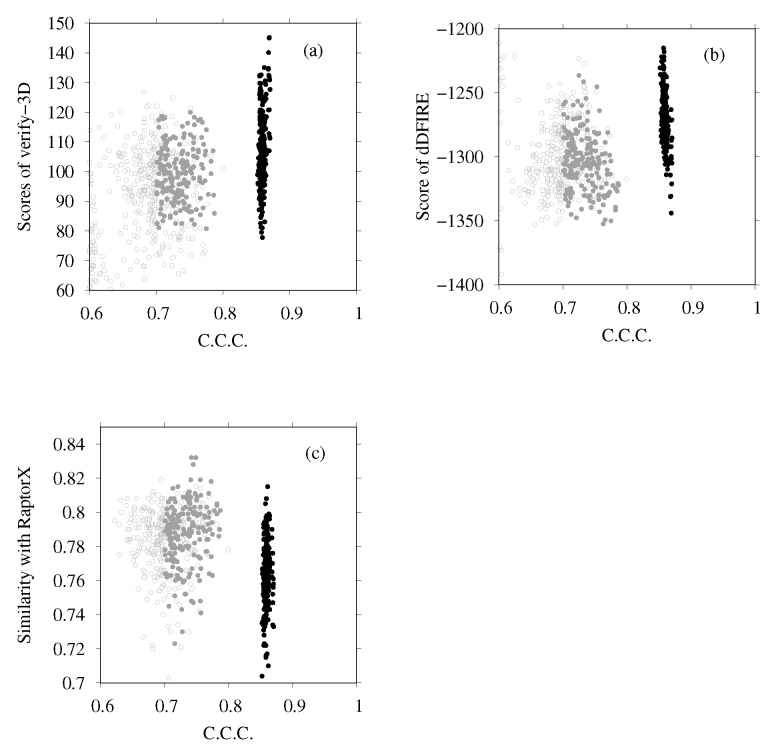
(**a**) Scores of VERIFY3D vs. CCC calculated using the colores of homology models before and after flexible-fitting simulations of the a-subunit. (**b**) Scores of dDFIRE vs. CCC calculated using the colores of homology models before and after flexible-fitting simulations of the a-subunit. (**c**) Similarity with secondary structure prediction by RaptorX Property vs. CCC calculated using the colores of homology models before and after flexible-fitting simulations of a-subunit. For each figure, the gray points represent homology models before flexible fitting. The filled dark-gray points represent the models selected for flexible fitting. The black points represent models after flexible fitting.

Next we examine the results of homology modeling for E-, G-, a- and d-subunits, for which structures are unknown (E- and G-subunits belong to the V${}_{1}$ region, and a- and d-subunits to the V${}_{0}$ region). The whole EM map was split into fragments corresponding to two EG complexes (stalk A and stalk B), an a-subunit, and a d-subunit. The gray points in Figure 8a,b are scattered points of model quality scores and the CCC of stalk A. In addition, Figure 8c,d show those of stalk B. Each point represents one complex structure. To construct these EG-complex structures, we performed homology modeling of the E-subunit and G-subunit. Then, based on the scores of similarity with the secondary structure prediction by RaptorX Property, the model quality scores were calculated using VERIFY3D, dDFIRE, and the Z-score of FORTE, and we selected the numbers of structures for each subunit (45 for the E-subunit and 35 for the G-subunit). The EG-complex structures were composed of selected subunits by superposition on the structure of PDB ID code 5Y5X. Structures with a higher CCC (CCC > 0.75) were selected for additional flexible-fitting simulation. Gray points in Figure 9 and Figure 10 present the results of the homology modeling of the d- and a-subunits. For both figures, the X-axes show the CCC with corresponding fragments of the EM map and the Y-axes show scores of model quality or similarity with secondary structure prediction. For the d-subunit, models with higher scores (CCC > 0.75 and similarity with secondary prediction >0.75, and the score of VERIFY3D >80) were selected for the next flexible-fitting simulation. For the a-subunit, models with higher scores (CCC >0.7 and similarity with secondary prediction >0.7, and the score of VERIFY3D >80) were selected for the next flexible-fitting simulation. Then, the results of the flexible-fitting simulations for each selected model of stalks A and B, the d-subunit, and the a-subunit were examined. The changes in the scores are presented as different positions of the black points in Figure 8, Figure 9 and Figure 10. In each figure, the gray points represent the scores of models generated by homology modeling. The black points show the scores of models obtained from the selected models after flexible-fitting simulations. All figures show that the CCC increases without loss of model quality.

**Figure 11:**
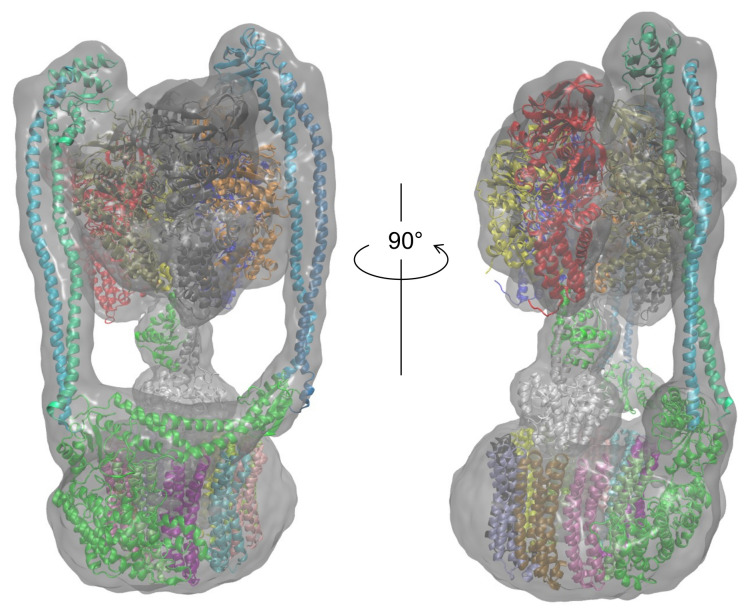
Entire atomic structure model of *Eh* V-ATPase in the EM map.

**Figure 12:**
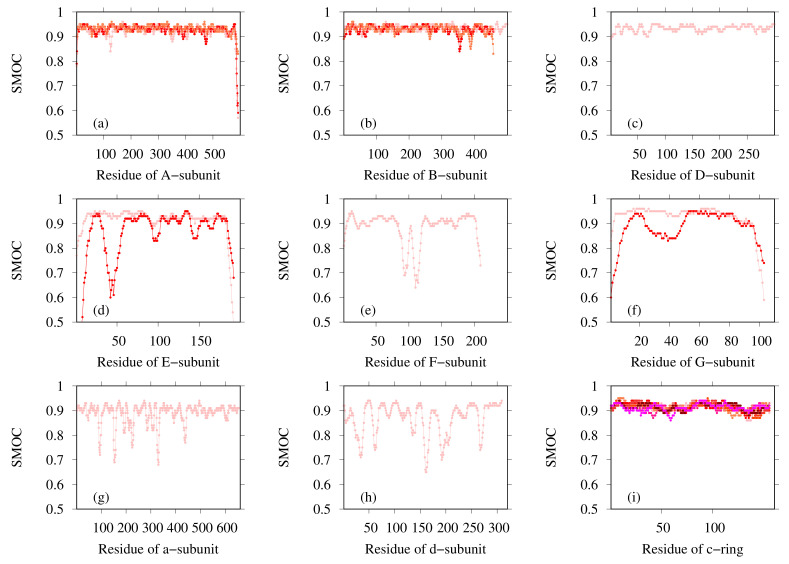
SMOC calculated using TEMPy for our model for (**a**) A-subunit, (**b**) B-subunit, (**c**) D-subunit, (**d**) E-subunit, (**e**) F-subunit, (**f**) G-subunit, (**g**) a-subunit, (**h**) d-subunit, and (**i**) c-subunit. Lines of different colors represent different chains for subunits of the same type.

Finally, the most probable model for each subunit was selected based on the CCC and model quality scores. Table 3 presents the PDB ID codes of the selected PDB structures for structurally known subunits and the templates for structurally unknown subunits. These subunits were assembled for the entire structure of *Eh* V-ATPase. Finally, the flexible-fitting simulation was performed for the entire structure (Figure 11). We compare our modeling with the earlier discussion of global structure modeling based on cryo-EM data with lower resolution [46]. An earlier study also used methods that adopt homology modeling. As the template structures used for the modeling of structurally unknown subunits, the same PDB was used for the d-subunit; different PDB structures were used for the a-, E-, and G-subunits in this study. Regarding the a-subunit, however, the PDB for the same protein, *Thermus thermophilus*, has been updated from 5GAR [80] to 5Y5X. For the E-subunit and G-subunit, distantly related proteins such as 4DT0 (subunit E of *Pyrococcus horikoshii* OT3 A-ATP synthase) [81], 3DHR (pigeon methemoglobin) [82], and 2XNX (BC1 fragment of streptococcal M1 protein) were found through profile–profile analysis. Models based on these templates were selected as better models than other generated models by their agreement with the EM map calculated using colores. The values related to goodness of fit to the EM map are presented in Table 4. For metrics of all types, the values are rather high, especially for correlation calculated using Chimera (0.901) and SMOC (0.908). The SMOCs of each chain per residue are also shown in Figure 12. This figure shows that the final model fundamentally fits the EM map of the overall structure well, although the models obtained using our method have some regions to be improved.

## 4. Discussion and Conclusions

Our developed homology modeling method is combined with cross-correlation-based flexible fitting to establish a method of constructing an atomic model based on an EM density map. Our homology modeling method is characterized by the adoption of enhanced profile–profile alignment in which our developed aligner, FORTE, is applied for alignment and comparison between profiles of templates and target proteins calculated in multiple ways. Due to FORTE-based alignment features, our homology modeling is expected to provide better initial structures for flexible-fitting simulation.

Two examples of application of our modeling method using the cryo-EM data were presented herein. In the first example, the dimer structure of human TPC2, one target of CASP13, was modeled. The structure of this target had already been determined using an EM map with 3.5 Å resolution [45]. This case is regarded as a demonstration of our method’s performance for the generation of initial structures for flexible-fitting simulation. The output models after flexible fitting closely approximate the reference structure in terms of TM-score. Moreover, the output models show very good fit to the EM map in terms of CC${}_{\mathrm{mask}}$ and CC${}_{\mathrm{peaks}}$, even though our five submitted models were not evaluated and filtered using the EM map before fitting in this case. Our models demonstrated superiority to almost all other models submitted in CASP13 in terms of the fit to the EM map and similarity to the reference structure after the same flexible-fitting simulation procedure. However, even for the model with the highest CC${}_{\mathrm{mask}}$, the CC-related values of our model were somewhat worse than those of the reference PDB structure. Considering the local fit to the EM map by SMOC per residue, it was found that the cause was in the loop and near-loop regions originating from the template structures of our homology modeling. These points were also observed in another example, i.e., modeling of the a-subunit of *Thermus thermophilus* V/A-ATPase (*T. thermophilus* V-ATPase). This target was also elucidated structurally using cryo-EM techniques with multiple resolutions [76,80,83]. Our model based on the EM map with 5.0 Å resolution showed better agreement with the EM map than the reference PDB structure (Table S1), and the SMOC of our model also showed lower values in a loop region (Figure S1). These modeling results demonstrate that it is often possible that our model does not fully fit a loop region to the EM map. To overcome this difficulty, we might be able to improve our method by considering partial adoption of de novo modeling or by increasing the bias to the EM map when using flexible-fitting simulation, only in areas for regions with a lower SMOC value.

As a second example in this paper and as a practical demonstration, construction of the entire structure of *Eh* V-ATPase was shown. In fact, *Eh* V-ATPase consists of 24 chains with 9 types of subunits. We constructed a reasonable model both in terms of fitting with the EM map and of model quality scores. It is noteworthy that the step of splitting the EM map into fragments was important for these models. Suitable fragments are useful for distinguishing between good and poor homology models. They often require manual tuning and repeated trials to partition the map properly. As already described in Section 2.1, it might be necessary to decide how to split the target sequence or to use multiple alignment for better homology modeling. Automating these processes or setting clear guidelines provides room for development of this method.

The novelty and strength of our method lies in a combination of enhanced profile–profile analyses and a filtering/fitting method using cryo-EM data. Profile–profile alignment enables us to create a group of accurate alignments based on the sensitive search for the template library including distantly related alignments. Filtering with the correlation with the cryo-EM map enables us to select the appropriate alignment (model) among them. Flexible-fitting simulation biased to the EM map used was sufficiently powerful to raise a low CC-related value to a high CC-related value for models generated by our homology modeling. We expect that if homology modeling is self-sufficient based on scores within the method (Z-score of FORTE and scores of model quality), then the final modeling is likely to be successful. Although few examples are presented herein, diversity does exist, as represented by homodimers and heterooligomers, and by those using multiple templates. It was possible to present some useful features and results of this method. In fact, this method is expected to play a valuable role in determining heterooligomer structures when the target EM data resolution is medium to low, and when the structures are outside the scope of ordinary de novo modeling methods.

Finally, we described the effects of recent progress that has been made in AI-based modeling of protein structures: as described in the Introduction, AF2, today’s state-of-the-art AI-based structure prediction method, achieved a breakthrough for the modeling of a monomer structure. However, multimer predictions based on AF2 [33,34,35] do not show a performance that is as good as the monomer prediction of AF2. Furthermore, it is expected that there are cases in which application of AF2 is not straightforward, not only because of the difficulty posed by multimers but also because AF2 is designed to generate only one state of a protein [84,85]. Additionally, we performed the modeling of TPC2 based on the model generated by AF2. The details are shown in the Supporting Information. These results indicate that even the state-of-the-art method requires flexible-fitting simulation to improve the goodness of fit to an EM map and that even then it might not reach a model (6NQ1 in this case) obtained by manually adjusted modeling using Coot [86] based on an EM map with high resolution in terms of local fitting. We therefore expect that, as long as these frontiers remain, some room exists for the combination of template-based modeling and flexible-fitting simulation to be useful for the construction of models using cryo-EM data.

## Figures and Tables

**Table 1:** Summary of methods used for profile generation: “nr”and “CDD” stand for the NCBI nr database and the Conserved Domain Database, respectively. Numbers in () represent the numbers of iterations.

	Method
(1)	SSEARCH(nr,MIQS) + MAFFT + PSI-BLASTexB(nr,3)
(2)	DELTA-BLAST(CDD)
(3)	HHblits(Uniclust) + PSI-BLASTexB(nr,1)

**Table 2:** Scores of 6NQ1 and of our model of T0984o for CASP13: CC${}_{\mathrm{mask}}$, CC${}_{\mathrm{volume}}$, CC${}_{\mathrm{peaks}}$, and CC${}_{\mathrm{box}}$ were calculated using the map_model_cc module in PHENIX. Correlation was calculated using the fit_in_map module in Chimera. The value of SMOC over every residue was calculated based on the values obtained using TEMPy.

	6NQ1	Our Model
CC${}_{\mathrm{mask}}$	0.823	0.755
CC${}_{\mathrm{volume}}$	0.794	0.713
CC${}_{\mathrm{peaks}}$	0.516	0.468
CC${}_{\mathrm{box}}$	0.585	0.557
CCC	0.593	0.566
Correlation (fit_in_map)	0.858	0.829
SMOC (avg.)	0.850	0.811
TM-score	1.0	0.930

**Table 3:** Summary of PDB IDs of the selected PDB structures for structurally known subunits and templates for structurally unknown subunits.

Subunit	Template (PDB ID(s))	Method (Resolution)
a-subunit	5Y5X	EM (5.0 Å)
c-ring	2BL2	X-ray (2.1 Å)
d-subunit	1R5Z	X-ray (2.05 Å)
E-subunit (Stalk A)	4DT0	X-ray (3.65 Å)
G-subunit (Stalk A)	3DHR	X-ray (2.0 Å)
E-subunit (Stalk B)	4DT0	X-ray (3.65 Å)
G-subunit (Stalk B)	2XNX	X-ray (3.3 Å)
A3B3-complex	5KND	X-ray (2.89 Å)
DF-complex	5KND/3AON	X-ray (2.89 Å/2.0 Å)

**Table 4:** CC-related values of the model of *Eh* V-ATPase: CC${}_{\mathrm{mask}}$, CC${}_{\mathrm{volume}}$, CC${}_{\mathrm{peaks}}$, and CC${}_{\mathrm{box}}$ were calculated using the map_model_cc module of PHENIX. Correlation was calculated using the fit_in_map module in Chimera. The value of SMOC over every residue was calculated based on values obtained using TEMPy.

	Our Model
CC${}_{\mathrm{mask}}$	0.652
CC${}_{\mathrm{volume}}$	0.534
CC${}_{\mathrm{peaks}}$	0.352
CC${}_{\mathrm{box}}$	0.892
CCC	0.386
Correlation (fit_in_map)	0.901
SMOC (avg.)	0.908

## Data Availability

The code of PSI-BLASTexB is available at GitHub, https://github.com/kyungtaekLIM/PSI-BLASTexB. FORTE, MAFFT, and SSEARCH with MIQS are available at http://forteprtl.cbrc.jp/forte/ (under refit), https://mafft.cbrc.jp/alignment/server/ and http://csas.cbrc.jp/Ssearch/. Density maps of *Eh* V-ATPase have been deposited in the EMDB with accession codes EMD-32034 (structure of unmodified *Eh* V-ATPase using Volta phase plate).

## Supplementary Materials

The following supporting information can be downloaded at: https://www.mdpi.com/article/10.3390/ijms23041977/s1, Analyses of modeling of *T. thermophilus* V-ATPase by our method and of human TPC2 based on the model generated by AlphaFold2 are provided as Supporting Information. The alignments used for the models of human TPC2, a-subunit of *T. thermophilus* V-ATPase, and subunits of *Eh* V-ATPase, which are not yet structurally determined, are also provided in the Supporting Information.

## References

[1] Kühlbrandt W. (2014). Cryo-EM enters a new era. eLife.

[2] Bai X.C., Fernandez I.S., McMullan G., Scheres S.H.W. (2013). Ribosome structures to near-atomic resolution from thirty thousand cryo-EM particles. eLife.

[3] Bai X.C., McMullan G., Scheres S.H.W. (2015). How cryo-EM is revolutionizing structural biology. Trends Biochem. Sci..

[4] Yip K.M., Fischer N., Paknia E., Chari A., Stark H. (2020). Atomic-resolution protein structure determination by cryo-EM. Nature.

[5] Takeda H., Tsutsumi A., Nishizawa T., Lindau C., Busto J.V., Wenz L.S., Ellenrieder L., Imai K., Straub S.P., Mossmann W. (2021). Mitochondrial sorting and assembly machinery operates by *β*-barrel switching. Nature.

[6] Fisette O., Schröder G.F., Schäfer L.V. (2020). Atomistic structure and dynamics of the human MHC-I peptide-loading complex. Proc. Natl. Acad. Sci. USA.

[7] Maity K., Heumann J.M., McGrath A.P., Kopcho N.J., Hsu P.K., Lee C.W., Mapes J.H., Garza D., Krishnan S., Morgan G.P. (2019). Cryo-EM structure of OSCA1.2 from Oryza sativa elucidates the mechanical basis of potential membrane hyperosmolality gating. Proc. Natl. Acad. Sci. USA.

[8] Zhang M., Gui M., Wang Z.-F., Gorgulla C., Yu J.J., Wu H., Sun Z.-Y.J., Klenk C., Merklinger L., Morstein L. (2021). Cryo-EM structure of an activated GPCR–G protein complex in lipid nanodiscs. Nat. Struct. Mol. Biol..

[9] Kryshtafovych A., Malhotra S., Monastyrskyy B., Cragnolini T., Joseph A.P., Chiu W., Topf M. (2019). Cryo-electron microscopy targets in CASP13: Overview and evaluation of results. Proteins Struct. Funct. Bioinf..

[10] Baker M.R., Rees I., Ludtke S.J., Chiu W., Baker M.L. (2012). Constructing and validating initial C*α* models from subnanometer resolution density maps with pathwalking. Structure.

[11] Chen M., Baldwin P.R., Ludtke S.J., Baker M.L. (2016). De Novo modeling in cryo-EM density maps with Pathwalking. J. Struct. Biol..

[12] Dimaio F., Song Y., Li X., Brunner M.J., Xu C., Conticello V., Egelman E., Marlovits T.C., Cheng Y., Baker D. (2015). Atomic-accuracy models from 4.5-Å cryo-electron microscopy data with density-guided iterative local refinement. Nat. Methods.

[13] Wang R.Y.R., Kudryashev M., Li X., Egelman E.H., Basler M., Cheng Y., Baker D., Dimaio F. (2015). De novo protein structure determination from near-atomic-resolution cryo-EM maps. Nat. Methods.

[14] Terashi G., Kihara D. (2018). De novo main-chain modeling for em maps using MAINMAST. Nat. Commun..

[15] Gao H., Sengupta J., Valle M., Korostelev A., Eswar N., Stagg S.M., Van Roey P., Agrawal R.K., Harvey S.C., Sali A. (2003). Study of the structural dynamics of the E. coli 70S ribosome using real-space refinement. Cell.

[16] Velazquez-Muriel J.A., Carazo J.M. (2007). Flexible fitting in 3D-EM with incomplete data on superfamily variability. J. Struct. Biol..

[17] Gorba C., Miyashita O., Tama F. (2008). Normal-mode flexible fitting of high-resolution structure of biological molecules toward one-dimensional low-resolution data. Biophys. J..

[18] Zhu J., Cheng L., Fang Q., Zhou Z.H., Honig B. (2010). Building and Refining Protein Models within Cryo-electron Microscopy Density Maps Based on Homology Modeling and Multiscale Structure Refinement. J. Mol. Biol..

[19] Kirmizialtin S., Loerke J., Behrmann E., Spahn C.M.T., Sanbonmatsu K.Y. (2015). Using molecular simulation to model high-resolution cryo-EM reconstructions. Methods Enzymol..

[20] Singharoy A., Teo I., McGreevy R., Stone J.E., Zhao J., Schulten K. (2016). Molecular dynamics-based refinement and validation for sub-5 Å cryo-electron microscopy maps. eLife.

[21] Kim D.N., Moriarty N.W., Kirmizialtin S., Afonine P.V., Poon B., Sobolev O.V., Adams P.D., Sanbonmatsu K. (2019). Cryo-fit: Democratization of flexible fitting for cryo-EM. J. Struct. Biol..

[22] Afonine P.V., Poon B.K., Read R.J., Sobolev O.V., Terwilliger T.C., Urzhumtsev A., Adams P.D. (2018). Real-space refinement in PHENIX for cryo-EM and crystallography. Acta Crystallogr. D.

[23] Trabuco L.G., Villa E., Mitra K., Frank J., Schulten K. (2008). Flexible Fitting of Atomic Structures into Electron Microscopy Maps Using Molecular Dynamics. Structure.

[24] Trabuco L.G., Villa E., Schreiner E., Harrison C.B., Schulten K. (2009). Molecular dynamics flexible fitting: A practical guide to combine cryo-electron microscopy and X-ray crystallography. Methods.

[25] phenix.cryo_fit2 webpage. https://www.phenix-online.org/documentation/reference/dock_in_map.html.

[26] Ratje A.H., Loerke J., Mikolajka A., Brünner M., Hildebrand P.W., Starosta A.L., Dönhöfer A., Connell S.R., Fucini P., Mielke T. (2010). Head swivel on the ribosome facilitates translocation by means of intra-subunit tRNA hybrid sites. Nature.

[27] Jumper J., Evans R., Pritzel A., Green T., Figurnov M., Ronneberger O., Tunyasuvunakool K., Bates R., Žídek A., Potapenko A. (2021). Highly accurate protein structure prediction with AlphaFold. Nature.

[28] Jumper J., Evans R., Pritzel A., Green T., Figurnov M., Ronneberger O., Tunyasuvunakool K., Bates R., Žídek A., Potapenko A. (2021). Applying and improving AlphaFold at CASP14. Proteins Struct. Funct. Bioinf..

[29] Baek M., Anishchenko I., Park H., Humphreys I.R., Baker D. (2021). Protein oligomer modeling guided by predicted interchain contacts in CASP14. Proteins Struct. Funct. Bioinf..

[30] Lensink M.F., Brysbaert G., Mauri T., Nadzirin N., Velankar S., Chaleil R.A.G., Clarence T., Bates P.A., Kong R., Liu B. (2021). Prediction of protein assemblies, the next frontier: The CASP14-CAPRI experiment. Proteins Struct. Funct. Bioinf..

[31] Dapkūnas J., Olechnovič K., Česlovas V. (2021). Modeling of protein complexes in CASP14 with emphasis on the interaction interface prediction. Proteins Struct. Funct. Bioinf..

[32] Park T., Woo H., Yang J., Kwon S., Won J., Seok C. (2021). Protein oligomer structure prediction using GALAXY in CASP14. Proteins Struct. Funct. Bioinf..

[33] Evans R., O’Neill M., Pritzel A., Antropova N., Senior A., Green T., Žídek A., Bates R., Blackwell S., Yim J. (2021). Protein complex prediction with AlphaFold-Multimer. bioRxiv.

[34] Ghani U., Desta I., Jindal A., Khan O., Jones G., Kotelnikov S., Padhorny D., Vajda S., Kozakov D. (2021). Improved Docking of Protein Models by a Combination of Alphafold2 and ClusPro. bioRxiv.

[35] Bryant P., Pozzati G., Elofsson A. (2021). Improved prediction of protein-protein interactions using AlphaFold2 and extended multiple-sequence alignments. bioRxiv.

[36] Kryshtafovych A., Moult J., Albrecht R., Chang G.A., Chao K., Fraser A., Greenfield J., Hartmann M.D., Herzberg O., Josts I. (2021). Computational models in the service of X-ray and cryo-electron microscopy structure determination. Proteins Struct. Funct. Bioinf..

[37] Mosalaganti S., Obarska-Kosinska A., Siggel M., Turonova B., Zimmerli C.E., Buczak K., Schmidt F.H., Margiotta E., Mackmull M.T., Hagen W. (2021). Artificial intelligence reveals nuclear pore complexity. bioRxiv.

[38] Nakamura T., Oda T., Fukasawa Y., Tomii K. (2018). Template-based quaternary structure prediction of proteins using enhanced profile–profile alignments. Proteins Struct. Funct. Bioinf..

[39] Tomii K., Akiyama Y. (2004). FORTE: A profile-profile comparison tool for protein fold recognition. Bioinformatics.

[40] Tomii K., Hirokawa T., Motono C. (2005). Protein structure prediction using a variety of profile libraries and 3D verification. Proteins Struct. Funct. Bioinf..

[41] Lensink M.F., Velankar S., Kryshtafovych A., Huang S.Y., Schneidman-Duhovny D., Sali A., Segura J., Fernandez-Fuentes N., Viswanath S., Elber R. (2016). Prediction of homoprotein and heteroprotein complexes by protein docking and template-based modeling: A CASP-CAPRI experiment. Proteins Struct. Funct. Bioinf..

[42] Lensink M.F., Velankar S., Baek M., Heo L., Seok C., Wodak S.J. (2018). The challenge of modeling protein assemblies: The CASP12-CAPRI experiment. Proteins Struct. Funct. Bioinf..

[43] Shiota T., Imai K., Qiu J., Hewitt V.L., Tan K., Shen H.H., Sakiyama N., Fukasawa Y., Hayat S., Kamiya M. (2015). Molecular architecture of the active mitochondrial protein gate. Science.

[44] Kryshtafovych A., Moult J., Billings W.M., Corte D.D., Fidelis K., Kwon S., Olechnovič K., Seok C., Česlovas V., Won J. (2021). Modeling SARS-CoV-2 proteins in the CASP-commons experiment. Proteins Struct. Funct. Bioinf..

[45] She J., Zeng W., Guo J., Chen Q., Bai X.C., Jiang Y. (2019). Structural mechanisms of phospholipid activation of the human TPC2 channel. eLife.

[46] Tsunoda J., Song C., Imai F.L., Takagi J., Ueno H., Murata T., Iino R., Murata K. (2018). Off-axis rotor in Enterococcus hirae V-ATPase visualized by Zernike phase plate single-particle cryo-electron microscopy. Sci. Rep..

[47] Li W., Godzik A. (2006). Cd-hit: A fast program for clustering and comparing large sets of protein or nucleotide sequences. Bioinformatics.

[48] Fu L., Niu B., Zhu Z., Wu S., Li W. (2012). CD-HIT: Accelerated for clustering the next-generation sequencing data. Bioinformatics.

[49] Pearson W. (2000). Flexible sequence similarity searching with the FASTA3 program package. Methods Mol. Biol..

[50] Yamada K., Tomii K. (2014). Revisiting amino acid substitution matrices for identifying distantly related proteins. Bioinformatics.

[51] Nakamura T., Yamada K.D., Tomii K., Katoh K. (2018). Parallelization of MAFFT for large-scale multiple sequence alignments. Bioinformatics.

[52] Oda T., Lim K., Tomii K. (2017). Simple adjustment of the sequence weight algorithm remarkably enhances PSI-BLAST performance. BMC Bioinform..

[53] Altschul S.F., Madden T.L., Schäffer A.A., Zhang J., Zhang Z., Miller W., Lipman D.J. (1997). Gapped BLAST and PSI-BLAST: A new generation of protein database search programs. Nucleic Acids Res..

[54] Boratyn G.M., Schäffer A.A., Agarwala R., Altschul S.F., Lipman D.J., Madden T.L. (2012). Domain enhanced lookup time accelerated BLAST. Biol. Direct.

[55] Remmert M., Biegert A., Hauser A., Söding J. (2012). HHblits: Lightning-fast iterative protein sequence searching by HMM-HMM alignment. Nat. Methods.

[56] Mirdita M., von den Driesch L., Galiez C., Martin M.J., Söding J., Steinegger M. (2016). Uniclust databases of clustered and deeply annotated protein sequences and alignments. Nucleic Acids Res..

[57] Webb B., Sali A. (2016). Comparative protein structure modeling using MODELLER. Curr. Protoc. Bioinform..

[58] Lüthy R., Bowie J.U., Eisenberg D. (1992). Assessment of protein models with three-dimensional profiles. Nature.

[59] Eisenberg D., Lüthy R., Bowie J.U. (1997). VERIFY3D: Assessment of protein models with three-dimensional profiles. Methods Enzymol..

[60] Yang Y., Zhou Y. (2008). Ab initio folding of terminal segments with secondary structures reveals the fine difference between two closely related all-atom statistical energy functions. Protein Sci..

[61] Yang Y., Zhou Y. (2008). Specific interactions for ab initio folding of protein terminal regions with secondary structures. Proteins Struct. Funct. Bioinf..

[62] Wang S., Li W., Liu S., Xu J. (2016). RaptorX-Property: A web server for protein structure property prediction. Nucleic Acids Res..

[63] Wriggers W. (2012). Conventions and workflows for using Situs. Acta Crystallogr. D.

[64] Hess B., Kutzner C., van der Spoel D., Lindahl E. (2008). GROMACS 4: Algorithms for Highly Efficient, Load-Balanced, and Scalable Molecular Simulation. J. Chem. Theory Comput..

[65] Afonine P.V., Klaholz B.P., Moriarty N.W., Poon B.K., Sobolev O.V., Terwilliger T.C., Adams P.D., Urzhumtsev A. (2018). New tools for the analysis and validation of cryo-EM maps and atomic models. Acta Crystallogr. D.

[66] Manders E.M.M., Verbeek F.J., Aten J.A. (1993). Measurement of co-localization of objects in dual-colour confocal images. J. Micros..

[67] Farabella I., Vasishtan D., Joseph A.P., Pandurangan A.P., Sahota H., Topf M. (2015). *TEMPy*: A Python library for assessment of three-dimensional electron microscopy density fits. J. Appl. Crystallogr..

[68] She J., Guo J., Chen Q., Zeng W., Jiang Y., Bai X.C. (2018). Structural insights into the voltage and phospholipid activation of the mammalian TPC1 channel. Nature.

[69] Zhang Y., Skolnick J. (2004). Scoring function for automated assessment of protein structure template quality. Proteins Struct. Funct. Bioinf..

[70] Murata T., Yamato I., Kakinuma Y., Leslie A.G.W., Walker J.E. (2005). Structure of the rotor of the V-type Na+-ATPase from Enterococcus hirae. Science.

[71] Saijo S., Arai S., Hossain K.M.M., Yamato I., Suzuki K., Kakinuma Y., Ishizuka-Katsura Y., Ohsawa N., Terada T., Shirouzu M. (2011). Crystal structure of the central axis DF complex of the prokaryotic V-ATPase. Proc. Natl. Acad. Sci. USA.

[72] Arai S., Saijo S., Suzuki K., Mizutani K., Kakinuma Y., Ishizuka-Katsura Y., Ohsawa N., Terada T., Shirouzu M., Yokoyama S. (2013). Rotation mechanism of Enterococcus hirae v 1-ATPase based on asymmetric crystal structures. Nature.

[73] Suzuki K., Kimura T., Shinoda H., Bai G., Daniels M.J., Arai Y., Nakano M., Nagai T. (2016). Five colour variants of bright luminescent protein for real-time multicolour bioimaging. Nat. Commun..

[74] Pettersen E.F., Goddard T.D., Huang C.C., Couch G.S., Greenblatt D.M., Meng E.C., Ferrin T.E. (2004). UCSF Chimera - A visualization system for exploratory research and analysis. J. Comput. Chem..

[75] Pintilie G.D., Zhang J., Goddard T.D., Chiu W., Gossard D.C. (2010). Quantitative analysis of cryo-EM density map segmentation by watershed and scale-space filtering, and fitting of structures by alignment to regions. J. Struct. Biol..

[76] Nakanishi A., Kishikawa J., Tamakoshi M., Mitsuoka K., Yokoyama K. (2018). Cryo EM structure of intact rotary H+-ATPase/synthase from Thermus thermophilus. Nat. Commun..

[77] Williams C.J., Headd J.J., Moriarty N.W., Prisant M.G., Videau L.L., Deis L.N., Verma V., Keedy D.A., Hintze B.J., Chen V.B. (2018). MolProbity: More and better reference data for improved all-atom structure validation. Protein Sci..

[78] Mukherjee S., Zhang Y. (2009). MM-align: A quick algorithm for aligning multiple-chain protein complex structures using iterative dynamic programming. Nucleic Acids Res..

[79] Mizutani K., Yamamoto M., Suzuki K., Yamato I., Kakinuma Y., Shirouzu M., Walker J.E., Yokoyama S., Iwata S., Murata T. (2011). Structure of the rotor ring modified with N,N’-dicyclohexylcarbodiimide of the Na+-transporting vacuolar ATPase. Proc. Natl. Acad. Sci. USA.

[80] Schep D.G., Zhao J., Rubinstein J.L. (2016). Models for the a subunits of the Thermus thermophilus V/A-ATPase and Saccharomyces cerevisiae V-ATPase enzymes by cryo-EM and evolutionary covariance. Proc. Natl. Acad. Sci. USA.

[81] Balakrishna A.M., Hunke C., Grüber G. (2012). The Structure of Subunit E of the Pyrococcus horikoshii OT3 A-ATP Synthase Gives Insight into the Elasticity of the Peripheral Stalk. J. Mol. Biol..

[82] Macheboeuf P., Buffalo C., Fu C.Y., Zinkernagel A.S., Cole J.N., Johnson J.E., Ghosh V.N.P. (2011). Streptococcal M1 protein constructs a pathological host fibrinogen network. Nature.

[83] Zhou L., Sazanov L.A. (2019). Structure and conformational plasticity of the intact Thermus thermophilus V/A-type ATPase. Science.

[84] Heo L., Feig M. (2021). Multi-State Modeling of G-protein Coupled Receptors at Experimental Accuracy. bioRxiv.

[85] del Alamo D., Sala D., Mchaourab H.S., Meiler J. (2021). Sampling the conformational landscapes of transporters and receptors with AlphaFold2. bioRxiv.

[86] Emsley P., Cowtan K. (2004). *Coot*: Model-building tools for molecular graphics. Acta Crystallogr. D.

