# Supplementary material for: Application of Homology Modeling by Enhanced Profile–Profile Alignment and Flexible-Fitting Simulation to Cryo-EM Based Structure Determination
Source: Int J Mol Sci. 2022 Feb 10;23(4):1977. doi: 10.3390/ijms23041977 (PMC8879198; doi:10.3390/ijms23041977)
Supplement: Supplementary file 1 [file ijms-23-01977-s001.zip › ijms-1536967-supplementary.pdf]

Supporting Information

1. Modeling of the a-subunit of Thermus thermophilus V/A-ATPase

As another demonstration of the performance which can be achieved using our method, modeling of the a-subunit of *Thermus thermophilus* V/A-ATPase (*T. thermophilus* V-ATPase) was performed based on currently available EM maps. The overall structure of *T. thermophilus* V-ATPase has been ascertained already using cryo-EM techniques with multiple resolution. We compared the existing PDB structure (reference structure) with the model generated from the viewpoint of fitness with EM-maps. Among the known structures of the a-subunit, the chain N of 5Y5X (PDB ID code), which was found based on EM map (EMD-6810) with 5.0 Å resolution [1], was selected as the reference structure. This choice was executed because our method specifically examines modeling based on EM data with medium resolution. First, the sequence of the a-subunit of *T. thermophilus* V-ATPase (UniProt accession number: Q5SIT6) was divided into two regions: residues of 1–310 (domain I) and residues of 311–652 (domain II). Our homology modeling pipeline was applied to domains I and II without information of these known structures. The most recent data of the used template were published in January 2018. The PDB structures for the 2016 paper [2] were also excluded from the template database. After scoring and ranking the models generated for each domain, the alignments corresponding to the high-scoring models were selected for both domains. Then, full-length models were newly generated with a chimeric template based on selected alignment combinations using MODELLER, with addition of secondary structure restraint based on prediction of RaptorX-property. The templates at this stage were unique for their respective domains: 3RRK for domain I and 5TJ5 for domain II. Then, whole models were subjected again to scoring and ranking based on the structure score and the CCC calculated using Colores to the EM map. Next, the higher-ranked models were subjected to the same procedure with the case of human TPC2 of flexible fitting using cryo_fit2 and evaluation. The ranges of TM-score for higher-ranked structures, which were up to 0.7, directly reflect that structure models generated by the pipeline are similar to the reference structure. However, the CCC values are still low (up to about 0.4 at most), even for structure models with a high TM-score, indicating a need for a flexible-fitting simulation. Finally, the model with the highest values of CC_{mask} was compared with the reference structure (Table S1). The SMOCs per residue were also calculated for both the reference structure and for our model (Figure S1). Values in the table and figure show that our model approaches the reference structure in terms of the TM-score and CCC. From the viewpoint of fitting to the EM map, our model shows improvement for CC-related values. Consideration of Figure S1 indicates that our model is superior to the reference structure in terms of fit to the EM map, except for the residues in regions such as 270-276, and 420–440. These areas are loop regions highlighted in Figure S2.

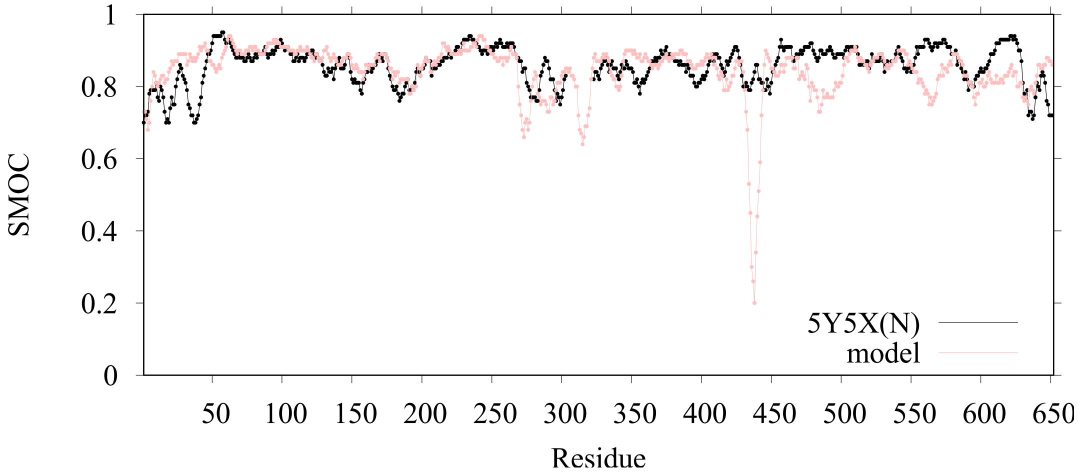

Figure S1. SMOC calculated using TEMPy for 5Y5X and our model: black corresponds to 5Y5X; pink corresponds to our model.

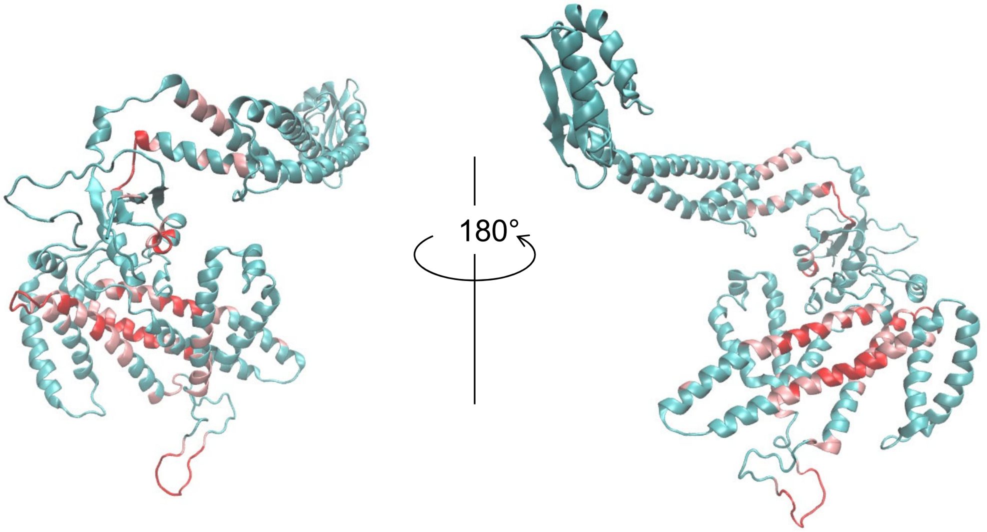

Figure S2. Our model of a-subunit of *T. thermophilus* V-ATPase, the region in which our model SMOC values are lower than PDB structure is highlighted (red regions are lower than pink).

Table S1. Scores of 5Y5X and our model of a-subunit of *T. thermophilus* V-ATPase: CC_{mask}, CC_{volume}, CC_{peaks}, and CC_{box} were calculated using the map_model_cc module in Phenix. Correlation was calculated using the fit_in_map module in Chimera. The value of SMOC for every residue was calculated based on values obtained using TEMPy.

	5Y5X	model
CC_mask	0.4220	0.5803
CC_peak	-0.1252	-0.1318
CC_volume	0.4900	0.5814
CC_box	0.2718	0.3437
Correlation (fit in map)	0.841	0.847
CCC	0.270	0.473
SMOC (avg.)	0.857	0.844
TMscore with 5Y5X	1.000	0.7542

References

1. Nakanishi, A.; Kishikawa, J.; Tamakoshi, M.; Mitsuoka, K.; Yokoyama, K. Cryo EM structure of intact rotary H+-ATPase/synthase from *Thermus thermophilus*. *Nat. Commun.* **2018**, *9*, 89.
2. Schep, D. G.; Zhao, J.; Rubinstein, J.L. Models for the a subunits of the *Thermus thermophilus* V/A-ATPase and *Saccharomyces cerevisiae* V-ATPase enzymes by cryo-EM and evolutionary covariance. *Proc. Natl. Acad. Sci.* **2016**, *113*, 3245–3250.

2. Comparison with a model generated using AlphaFold2

In support of our discussion, we present the modeling of AlphaFold2(AF2) for the same target of our method in the sense of global and local fitness to the EM map. For human TPC2, we performed AF2 modeling using AF2 ver. 2.00 and compared how the model fits to the EM map and how it is similar to the reference structure (PDB ID code: 6NQ1). The parameters of AF2 used for obtaining the monomer of TPC2 were "–max_template_date 2018-05-01" and –preset "full_dbs". We duplicated the monomer for building the homodimer based on the location of the PDB structure 6NQ1. This setting can be a favorable condition for the AF2 model (and might be concessional for us) because knowledge after the structure determination was used for the placement of two AF2-generated models. Then the same procedures of the flexible-fitting simulation and analyses were applied to the AF2 model. The values of goodness-of-fit to the EM map and of similarity with the PDB structure of the AF2 model after flexible fitting were found to be comparable with ours, as presented in Table S2. The local fitness measured as the SMOC value is also shown as the following Figure S3. Regions in which the SMOC value of the AF2 model after flexible fitting is lower than the value of the reference structure (such as residue is 315 to 330 for both chains) are highlighted in Figure S4. These results indicate that even the state-of-the-art approach requires flexible-fitting simulation to improve goodness-of-fit to an EM map. It might not reach a model (6NQ1 in this case) obtained by manually adjusted modeling using Coot based on an EM map with high-resolution. This finding implies that model-building into cryo-EM maps, especially for protein complexes remains as a frontier.

Table S2. Scores of 6NQ1, our model and AF2 model (before/after flexible-fitting simulation) of T0984o for CASP13: CC_{mask}, CC_{volume}, CC_{peaks}, and CC_{box} were calculated using the map_model_cc module in PHENIX. Correlation was calculated using the fit_in_map module in Chimera. The value of SMOC over every residue was calculated based on the values obtained using TEMPy.

	**6NQ1**	**Our Model (No Fit.)**	**Our Model (Fit.)**	**AF2 Model**	**AF2 Model (Fit.)**
CC_{mask}	0.823	0.284	0.755	0.439	0.757
CC_{volume}	0.794	0.322	0.713	0.439	0.715
CC_{peak}	0.516	0.083	0.468	0.236	0.475
CC_{box}	0.585	0.300	0.557	0.398	0.560
Correlation(fit in map)	0.858	0.661	0.829	0.740	0.843
CCC	0.593	0.445	0.566	0.516	0.569
SMOC(ave.)	0.850	0.628	0.811	0.688	0.803
TMscore	1.000	0.916	0.930	0.957	0.982

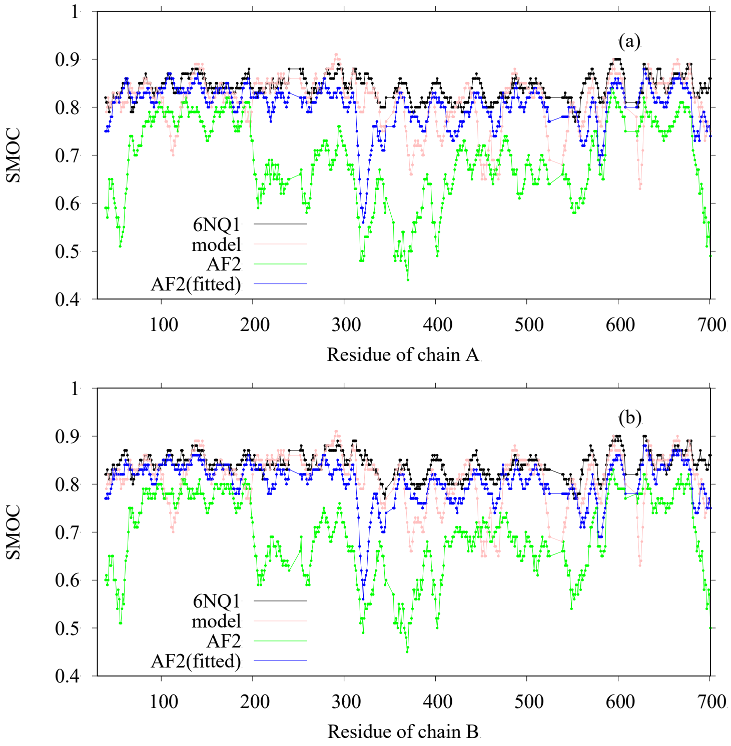

Figure S3. SMOC calculated using TEMPy for 6NQ1, our model, AF2 model and AF2 model after flexible-fitting simulation for (a) chain A and (b) chain B. Black corresponds to 6NQ1. Pink corresponds to our model. Green and blue lines respectively correspond to AF2 models before and after flexible fitting.

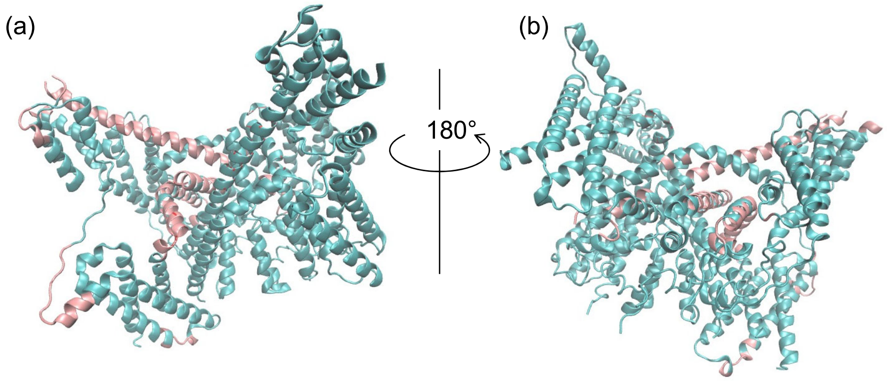

Figure S4. AlphaFold2-generated model after flexible-fitting simulation. Regions in which the SMOC value of AF2 model after flexible fitting is lower than the value of the reference structure are highlighted in pink.

Alignment used for the modeling of human TPC2

```
T0984   ------------------------------------------------------------------ARWDLCIDQAVVFIEDAIQY-RSINHRVDASSMWLYRRYYSNVCQRTLSFTIFLILFLAFIETPSSLTSTADVRYRAAPWEPPCGLTESVEVLCL

6C96    ------------------------------------------------------------------HNWEMNYQEAAIYLQEGQNNDKFFTHPKDARALAAYLFVHNHFFYMMELLTALLLLLLSLCESPA-----------VPVLKLHTYVHATLELFAL

6C9a    ------------------------------------------------------------------HNWEMNYQEAAIYLQEGQNNDKFFTHPKDARALAAYLFVHNHFFYMMELLTALLLLLLSLCESPA-----------VPVLKLHTYVHATLELFAL

T0984   LVFAADLSVKGYLFGWAHFQKNLWLLGYLVVLVVSLVDWTVSL--SLVCHEPLRIRRLLRPFFLLQNSSMMKKTLKCIRWSLPEMASVGLLLAIHLCLFTMFGMLLFAGGKQDDGQDRERLTYFQNLPESLTSLLVLLTTANNPDVMIPAYSKNRAYAIF

6C96    MVVVFELCMKLRWLGFHTFVRHKRTMVKTSVLVVQFIEAIVVLVRQTSHVRVTRALRCIFLVD-CRYCGGVRRNLRQIFQSLPPFMDILLLLLFFMIIFAILGFYLFST--------NPSDPYFSTLENSIVNLFVLLTTANFPDVMMPSYSRNPWSCVF

6C9a    MVVVFELCMKLRWLGFHTFVRHKRTMVKTSVLVVQFIEAIVVLVRQTSHVRVTRALRCIFLVD-CRYCGGVRRNLRQIFQSLPPFMDILLLLLFFMIIFAILGFYLFST--------NPSDPYFSTLENSIVNLFVLLTTANFPDVMMPSYSRNPWSCVF

T0984   FIVFTVIGSLFLMNLLTAIIYSQFRGYLMKSLQTSLFRRRLGTRAAFEVLSSMVGEGGAFPQAVGVKPQNLLQVLQ--KVQLDSSHKQAMMEKVRSYGSVLLSAEEFQKLFNELDRSVVKEH------PPRPEYQSPFLQSAQFLFGHYYFDYLGNLIAL

6C96    FIVYLSIELYFIMNLLLAVVFDTFNDIEKHKFKSLLLHKRTAIQHAYGLLASQRRPA-------GISYRQFEGLMRFYKPRMSARERFLTFKALNQSNTPLLSLKDFYDIYEVAALQWKAKRNRQHWFDELPRTAFLIFKGINILVNSKAFQYFMYLVVA

6C9a    FIVYLSIELYFIMNLLLAVVFDTFNDIEKHKFKSLLLHKRTAIQHAYGLLASQRRPA-------GISYRQFEGLMRFYKPRMSARERFLTFKALNQSNTPLLSLKDFYDIYEVAALQWKAKRNRQHWFDELPRTAFLIFKGINILVNSKAFQYFMYLVVA

T0984   ANLVSICVFLVLDADVLPAERDDFILG-ILNCVFIVYYLLEMLLKVFALGLRGYLSYPSNVFDGLLTVVLLVLEISTLAVYRLPHPGWRPEMVGLLSLWDMTRMLNMLIVFRFLRIIPSMKLMAVVASTVLGLVQNMRAFGGILVVVYYVFAIIGINLFR

6C96    VNGVWILVET----FMLKGGNFTSKHVPWSYLVFLTIYGVELFMKVAGLGPVEYLSSGWNLFDFSVTAFAFLGLLALTLNMEP---------------FYFIVVLRPLQLLRLFKLKKR---YRNVLDTMFELLPRMASLGLTLLTFYYSFAIVGMEFFN

6C9a    VNGVWILVET----FMLKGGNFTSKHVPWSYLVFLTIYGVELFMKVAGLGPVEYLSSGWNLFDFSVTAFAFLGLLALTLNMEP---------------FYFIVVLRPLQLLRLFKLKKR---YRNVLDTMFELLPRMASLGLTLLTFYYSFAIVGMEFFN

T0984   GVIVALPGNSSLAPANGSAPCGSF------EQLEYWANNFDDFAAALVTLWNLMVVNNWQVFLDAYRRYSGPWSKIYFVLWWLVSSVIWVNLFLALILENFL-----------------------------------------------------------

6C96    GRLTPNCCNTSTVADAYRFINHTVGNKTKVEEGYYYLNNFDNILNSFVTLFELTVVNNWYIIMEGVTSQTSHWSRLYFMTFYIVTMVVMT-IIVAFILEAFVFRMNYSRK-------SGIVIEKEMSKEELMAVLELYREERGTSSDVTRLLDTLSQMEK

6C9a    GRLTPNCCNTSTVADAYRFINHTVGNKTKVEEGYYYLNNFDNILNSFVTLFELTVVNNWYIIMEGVTSQTSHWSRLYFMTFYIVTMVVMT-IIVAFILEAFVFRMNYSRK-------SGIVIEKEMSKEELMAVLELYREERGTSSDVTRLLDTLSQMEK

T0984   ---------------------HKWDPR------------------------------------------------/

6C96    YQQNSMVFLGRRSRTKSDLSLKMYQEEIQEWYEEHAREQEQQKLRH------------------------------/

6C9a    YQQNSMVFLGRRSRTKSDLSLKMYQEEIQEWYEEHAREQEQQKLRH------------------------------/

T0984   ------------------------------------------------------------------ARWDLCIDQAVVFIEDAIQY-RSINHRVDASSMWLYRRYYSNVCQRTLSFTIFLILFLAFIETPSSLTSTADVRYRAAPWEPPCGLTESVEVLCL

6C96    -------------------------------------------------------------------NWEMNYQEAAIYLQEGQNNDKFFTHPKDARALAAYLFVHNHFFYMMELLTALLLLLLSLCESPA-----------VPVLKLHTYVHATLELFAL

6C9a    -------------------------------------------------------------------NWEMNYQEAAIYLQEGQNNDKFFTHPKDARALAAYLFVHNHFFYMMELLTALLLLLLSLCESPA-----------VPVLKLHTYVHATLELFAL

T0984   LVFAADLSVKGYLFGWAHFQKNLWLLGYLVVLVVSLVDWTVSL--SLVCHEPLRIRRLLRPFFLLQNSSMMKKTLKCIRWSLPEMASVGLLLAIHLCLFTMFGMLLFAGGKQDDGQDRERLTYFQNLPESLTSLLVLLTTANNPDVMIPAYSKNRAYAIF

6C96    MVVVFELCMKLRWLGFHTFVRHKRTMVKTSVLVVQFIEAIVVLVRQTSHVRVTRALRCIFLVD-CRYCGGVRRNLRQIFQSLPPFMDILLLLLFFMIIFAILGFYLFST--------NPSDPYFSTLENSIVNLFVLLTTANFPDVMMPSYSRNPWSCVF

6C9a    MVVVFELCMKLRWLGFHTFVRHKRTMVKTSVLVVQFIEAIVVLVRQTSHVRVTRALRCIFLVD-CRYCGGVRRNLRQIFQSLPPFMDILLLLLFFMIIFAILGFYLFST--------NPSDPYFSTLENSIVNLFVLLTTANFPDVMMPSYSRNPWSCVF

T0984   FIVFTVIGSLFLMNLLTAIIYSQFRGYLMKSLQTSLFRRRLGTRAAFEVLSSMVGEGGAFPQAVGVKPQNLLQVLQ--KVQLDSSHKQAMMEKVRSYGSVLLSAEEFQKLFNELDRSVVKEH------PPRPEYQSPFLQSAQFLFGHYYFDYLGNLIAL

6C96    FIVYLSIELYFIMNLLLAVVFDTFNDIEKHKFKSLLLHKRTAIQHAYGLLASQRRPA-------GISYRQFEGLMRFYKPRMSARERFLTFKALNQSNTPLLSLKDFYDIYEVAALQWKAKRNRQHWFDELPRTAFLIFKGINILVNSKAFQYFMYLVVA

6C9a    FIVYLSIELYFIMNLLLAVVFDTFNDIEKHKFKSLLLHKRTAIQHAYGLLASQRRPA-------GISYRQFEGLMRFYKPRMSARERFLTFKALNQSNTPLLSLKDFYDIYEVAALQWKAKRNRQHWFDELPRTAFLIFKGINILVNSKAFQYFMYLVVA

T0984   ANLVSICVFLVLDADVLPAERDDFILG-ILNCVFIVYYLLEMLLKVFALGLRGYLSYPSNVFDGLLTVVLLVLEISTLAVYRLPHPGWRPEMVGLLSLWDMTRMLNMLIVFRFLRIIPSMKLMAVVASTVLGLVQNMRAFGGILVVVYYVFAIIGINLFR

6C96    VNGVWILVET----FMLKGGNFTSKHVPWSYLVFLTIYGVELFMKVAGLGPVEYLSSGWNLFDFSVTAFAFLGLLALTLNMEP---------------FYFIVVLRPLQLLRLFKLKKR---YRNVLDTMFELLPRMASLGLTLLTFYYSFAIVGMEFFN

6C9a    VNGVWILVET----FMLKGGNFTSKHVPWSYLVFLTIYGVELFMKVAGLGPVEYLSSGWNLFDFSVTAFAFLGLLALTLNMEP---------------FYFIVVLRPLQLLRLFKLKKR---YRNVLDTMFELLPRMASLGLTLLTFYYSFAIVGMEFFN

T0984   GVIVALPGNSSLAPANGSAPCGSF------EQLEYWANNFDDFAAALVTLWNLMVVNNWQVFLDAYRRYSGPWSKIYFVLWWLVSSVIWVNLFLALILENFL-----------------------------------------------------------

6C96    GRLTPNCCNTSTVADAYRFINHTVGNKTKVEEGYYYLNNFDNILNSFVTLFELTVVNNWYIIMEGVTSQTSHWSRLYFMTFYIVTMVVMT-IIVAFILEAFVFRMNYSRK-------SGIVIEKEMSKEELMAVLELYREERGTSSDVTRLLDTLSQMEK

6C9a    GRLTPNCCNTSTVADAYRFINHTVGNKTKVEEGYYYLNNFDNILNSFVTLFELTVVNNWYIIMEGVTSQTSHWSRLYFMTFYIVTMVVMT-IIVAFILEAFVFRMNYSRK-------SGIVIEKEMSKEELMAVLELYREERGTSSDVTRLLDTLSQMEK

T0984   ---------------------HKWDPR------------------------------------------------

6C96    YQQNSMVFLGRRSRTKSDLSLKMYQEEIQEWYEEHAREQEQQKLR-------------------------------

6C9a    YQQNSMVFLGRRSRTKSDLSLKMYQEEIQEWYEEHAREQEQQKLR-------------------------------
```

Alignment used for modeling of the a-subunit of *T. thermophilus* V-ATPase

```
Target  ------MI---APMEKLVLAGPKGRAKELLQSLQQAGVVHLETLRPEALSAYQLSPEERAELRRWEAVSAGAEHTLSLLGLEAEPA-RPFPEGLEAAEKALSPIQAHAEGLTRQKQELEEELALAQAYLEPLERLAALAHG--LDKSPFLRVIPFLLTEK

3RRK    -------------MEKLIVAGPKRLARELLAELQKAGVVHIDPLRPDELGEYRLSPTEEAELKRWEAVVSQAEQSLTVVGLATVPSSKPFTGSLEEAEAVLRPVASRAEVLGKERAALEEEIQTIELFGKAAEKLAALAHGLDESPRLGVIPFLVAKPEE

5TJ5    ----------------------------------------------------------------------------------------------------------------------------------------------------------------

Target  ELPLVEEALRKALEDRYLLAHEAYAGGVAALVVVHRKEVDQAKAALSRAGVAELRLPGALGELPLSEAARRLKERAEAAPRELSEVRQHLAKLARESASTLQSLWTRAQDEVARLKALEELASGRFGFALLGYVPVKAKPKVEEALARHKESVVYAFEPV

3RRK    LEAVR-KALQEALADRFVLEAEPLENQLAALVVVKRSELEAARSSLSRLGLAELRFPGAYGAMPLGKAAARMKERARLAPEELVGIREEVARLSRESGEALIALWTRAKDEVARYKAVADMAAGKYGAALMGWVPQKAKGKVEEALGRLRDQIVY-----

5TJ5    ----------------------------------------------------------------------------------------------------------------------------------------------------------------

Target  DE------------------------------------HHEADRIPVVLDNPPWAKPFELLVSFLNT---------------------------------------------------------------------------------------------

3RRK    ----------------------------------------------------------------------------------------------------------------------------------------------------------------

5TJ5    -------------------------------------------------------------------XXXXXXXXXXXXXXXXXXXXXXXXXXXXXXXXXXXXXXXXXXXXXXXXXXXXXXXXXXXXXXXXXXXXXXXXXXXXXXXXXXXXXXXXXXXXXXXX

Target  ------------------------------------------------------------------------------------------------------------------------------------------------------------PKYG

3RRK    ----------------------------------------------------------------------------------------------------------------------------------------------------------------

5TJ5    XXXXXXXXXXXXXXXXXXXXXXXXXXXXXXXXXXXXXXXXXXXXXXXXXXXXXXXXXXXXXXXXXXXXXXXXXXXXXXXXXXXXXXXXXXXXXXXXXXXXXXXXXXXXXXXXXXXXXXXXXXXXXXXXXXXXXXXXXXXXXXXXXXXXXXXXXAQY

Target  TFDPTPVVPVFFPFWFGMIVGDIGYALLFYLVGRWLSGYVKRNEPLVIDLFALKLKPQVIGKLVHILNWMVFWTVVWGVIYGEFFGTFLEHLGVFGTP------------------EHPGLIPILIHRIDTAKTANLLILLSVAFGVVLVFFGLALRAYL

3RRK    ----------------------------------------------------------------------------------------------------------------------------------------------------------------

5TJ5    REINAGLPTIVTFPFMFAIMF---GHGFLMTLAALSLVLNEKKINK-----MKRGEIFDMAFTGRYIILLMGVFSMYTGFLYN-------------------------------------------GTENALLFSNSYKMKLSILMGFIHMTYSYFFSLA

Target  GLKHRHMAHFWEGVGYLGGLVGVLALAASYLGNLQAGWLQ------------------------------------------------GLMYLGFGVFLLAV---------------------------------------------------LMSRIWL

3RRK    ----------------------------------------------------------------------------------------------------------------------------------------------------------------

5TJ5    NHL------DIIGNFIPGLLFMQGIFGYLSVCIVYKWAVDWVKDGKPAPGLLNMLINMFLSPGTIDDELYPHQAKVQVFLLLMALVCIPWLLLVKPLHFKF---------------------------------------------------DIMIHQVI

Target  MIPEIFTQAGHILSHIRIYAVGAAGGILAGLLTDVGFALAERLG-----LLGVLLGLLVAGVLHLLILLLTTLGHMLQPIRLLWVEFFTKFGFYEENGRPYRPFKSVREA---

3RRK    ------------------------------------------------------------------------------------------------------------------

5TJ5    HTIEFCLNCVSHTASYLRLWALSLAHAQLSSVLWTMTIQIAFGFRGFVGVFMTVALFAMWFALTCAVLVLMEGTSAMLHSLRLHWVESMSK---------PYEPFAFEYKDMEV
```

Alignment used for the modeling of the a-subunit of *Eh* V-ATPase

```
Target  MAVTKMEKVTLISDKKNREILLQAVQGLHAVEIRDLFQESENNQWVETFFPEPEMIDKDKELAKLSYKLTDIRTAIQFIEHHGEKSQKKQHLKRRELSLDTLEKNYSE--EAFSKKLEEVLLLKEQWEQLVDERQQLEDQENWLLNWQNLDLAPKAFDSQ

5Y5XN   -MIAPMEKLVLAGPKGRAKELLQSLQQAGVVHLETLRPEALSAY--------QLSPEERAELRRWEAVSAGAEHTLSLLGLEAEP-------------ARPFPEGLEAAEKALSPIQAHAEGLTRQKQELEEELALAQAYLEPLERLAALAHGLDKSPFL

Target  MTKLVIGTVNAKNAESFKAEVAEINE-AYLEEINSSPTTTYFAYIVLRADESRMEEIASRYGFVKEDYLY---EGTPQQQLVAAKQSLQEIKDQQKKLSSAIGACSGYIKD-FEWTEEIFLARSEREAIKDRIIHTPYLILIQGWVDHEEKQELIHMLQN

5Y5XN   --RVIPFLLTEKELPLVEEALRKALEDRYLLAHEAYAGGVAALVVVHRKEVDQAKAALSRAGVAELRLPGALGELPLSEAARRLKERAEAAPRELSEVRQHLAKLARESASTLQSLWTRAQDEVARLKALEELASGRFGFALLGYVPVKAKPKVEEALAR

Target  ILASEEVYLTFDEPTDNEIAEEVPTKLKNHPIVAPFEMLTEMYSLPKYEEVDPTPWMMPFYLVFFGMMVADIGYGLLMFLGAFLLQKLVVLPRGM--------------QRFAKFFEILAIPSIIWGFIYSSFFGAALPKEIFGIHLPFPI--------L

5Y5XN   HKESV--VYAFEPVDEHHEADRIPVVLDNPPWAKPFELLVSFLNTPKYGTFDPTPVVPVFFPFWFGMIVGDIGYALLFYLVGRWLSGYVKRNEPLVIDLFALKLKPQVIGKLVHILNWMVFWTVVWGVIYGEFFGTFLEHLGVFGTPEHPGLIPILIHRI

Target  STTDDVNTILILSVIFGLIQILVGLFIAAKEHIKRKAYVDAVNDGFAWQGILLGIILILLGTMILKNNAFVYLGGALAVLSAVCILIIPVFQSSSKAKGIAKGAYNLYGLTGYIGDLVSYTRLMALGISGGSIAAAFNMLVAFMPPA---ARFSVGILLI

5Y5XN   DTAKTANLLILLSVAFGVVLVFFGLALRAYLGLKHRHMAHFW-EGVGYLGGLVGVLALAASYLGNLQAGWL-QGLMYLGFGVFLLAVL-----------MSRIWLMIPEIFTQAGHILSHIRIYAVGAAGGILAGLLTDVGFALAERLGLLGVLLGLLVA

Target  IVLQALNMFLTLLSAYVHGARLQYVEFFGK--FYTGGGRSFKPLKTVEKYVNINHKKK

5Y5XN   GVLHLLILLLTTLGHMLQPIRLLWVEFFTKFGFYEENGRPYRPFKSVREAQ-------
```

Alignment used for the modeling of the d-subunit of *Eh* V-ATPase

```
Target  --------------RELELISKDTFEQMIQTDSIDSLGEILQSTIYQPYIYDGFDKDFEANLSQERSKLFQWLKESAPE-
1r5z    --DDFAYLNARVRVRRGTLLKESFFQEALDL-SFADFLRLLSETVYGGELAGQGLPDVDRAVLRTQAKLVGDLPRLVTGE
Target  -PEIVWIYTMRYTFHNLKVLTKAEITGQNLDHLYIHDGFYSLEVLKDAIHTQVSVELPDSLM----DYIREVHEYCEEST
1r5z    AREAVRLLLLRNDLHNLQALLRAKATGRPFEEVLLLPGTLREEVWRQAYEAQDPAGMAQVLAVPGHPLARALRAVLRETQ
Target  ILQGIDVIYDRCFLTEQRRLGEQLGYPELLEEIIAFIDLTNITTTARGILQHRSAGFMTTVISSSGSI-PKDTLLSFVRG
1r5z    DLARVEALLAKRFFEDVAKAAKGLDQPALRDYLALEVDAENLRTAFKLQGSGLAPDAF---FLKGGRFVDRVRFARLMEG
Target  EMASFTQFLLTTDYSELLKQVIHEEQIDLVSLEQLKDDYLSSFYQVAQTQAFGPLPLLAFLNAKEVESKNLRLLIIGKRN
1r5z    D-YAVLDELSGTPFS-----GLSGVR-DLKALERGLRCVLLKEAKKGVQDPLGVGLVLAYVKEREWEAVRLRLLARRAYF
Target  HFSLEQLKERM---
1r5z    GLPRAQVEEEVVC-
```

Alignment used for the modeling of the E-subunit of *Eh* V-ATPase

```
Target  ---------------------------ERASFEEMKRKEIDQKFEVKKWQIEADFQKEKASKLEEIERSYRQLRNKQKMQ

4dt0    ---------------------------KIEYILNEARQQAEKIKEEARRNAEAKAEWIIRRAKTQAELEKQRIIANARLE

Target  VKQEILNAKQEVLQRLFTEATLQLENEPKEEQLALMKQMIQTLPI---NGTARLIPGEKSADILTPAVIAEWNEELP-FE

4dt0    VRRKRLAIQEEIISSVLEEVKRRLETMSEDEYFESVKALLKEAIKELNEKKVRVMSNEKTLGLIAS-RIEEIKSELGDVS

Target  LIREDFTEKAQAGLIID--DAGIQYNFLFSHLIKEIQETMSAEIAKELFD

4dt0    IEL-GETVDTMGGVIVETEDGRIRIDNTFEARMERFEGEIRSTIAKVLFG
```

Alignment used for the modeling of the G-subunit for stalk A of *Eh* V-ATPase

```
Target  -IKEAEENNQKKEEQVKAELAQYEQLKNNELIDLNKEFQERLTKLMKEKRKNEEEVTEDEQHKLELILEQFRQKVLEIEK

3hdr    KQALSEIETRHSEIIKLENSIRELHDMFMDMAMLVESQGEMIDRIEYNVEHAVDYVERAVSDTKKAVKYQSKARRKKIMI

Target  TYLEKEEEEIAKKKNLTNEII

3hdr    IICCVILGIIIASTIGGI---
```

Alignment used for the modeling of the G-subunit for stalk B of *Eh* V-ATPase

```
Target  ------------IKEAEENNQKKEEQVKAELAQYEQLKNNELIDLNKEFQERLTKLMKEKRKNEEEVTEDEQHKLELILE

2xnx    RLEKELEEKKEALELAIDQASRDYHRATALEKELEEKKKALELAIDQASQDYNRANVLEKELEAITREQEINRNLLGNAK

Target  QFRQKVLEIEKTYLEKEEEEIAKKKNLTNEII

2xnx    LELDQLSSEKEQLTIEKAKLEEEKQIS-----
```
